# Extreme Population Differences in the Human Zinc Transporter ZIP4 (SLC39A4) Are Explained by Positive Selection in Sub-Saharan Africa

**DOI:** 10.1371/journal.pgen.1004128

**Published:** 2014-02-20

**Authors:** Johannes Engelken, Elena Carnero-Montoro, Marc Pybus, Glen K. Andrews, Carles Lalueza-Fox, David Comas, Israel Sekler, Marco de la Rasilla, Antonio Rosas, Mark Stoneking, Miguel A. Valverde, Rubén Vicente, Elena Bosch

**Affiliations:** 1 Institute of Evolutionary Biology (CSIC-UPF), Department of Experimental and Health Sciences, Universitat Pompeu Fabra, Barcelona, Spain; 2 Department of Evolutionary Genetics, Max-Planck Institute for Evolutionary Anthropology, Leipzig, Germany; 3 Department of Biochemistry and Molecular Biology, University of Kansas Medical Center, Kansas City, Kansas, United States of America; 4 Department of Physiology, Ben-Gurion University, Beer-Sheva, Israel; 5 Área de Prehistoria, Departamento de Historia, Universidad de Oviedo, Oviedo, Spain; 6 Group of Paleoanthropology MNCN-CSIC, Department of Paleobiology, National Museum of Natural Sciences, CSIC, Madrid, Spain; 7 Laboratory of Molecular Physiology and Channelopathies, Department of Experimental and Health Sciences, Universitat Pompeu Fabra, Barcelona, Spain; University of Washington, United States of America

## Abstract

Extreme differences in allele frequency between West Africans and Eurasians were observed for a leucine-to-valine substitution (Leu372Val) in the human intestinal zinc uptake transporter, ZIP4, yet no further evidence was found for a selective sweep around the *ZIP4* gene (*SLC39A4*). By interrogating allele frequencies in more than 100 diverse human populations and resequencing Neanderthal DNA, we confirmed the ancestral state of this locus and found a strong geographical gradient for the derived allele (Val372), with near fixation in West Africa. In extensive coalescent simulations, we show that the extreme differences in allele frequency, yet absence of a classical sweep signature, can be explained by the effect of a local recombination hotspot, together with directional selection favoring the Val372 allele in Sub-Saharan Africans. The possible functional effect of the Leu372Val substitution, together with two pathological mutations at the same codon (Leu372Pro and Leu372Arg) that cause acrodermatitis enteropathica (a disease phenotype characterized by extreme zinc deficiency), was investigated by transient overexpression of human ZIP4 protein in HeLa cells. Both acrodermatitis mutations cause absence of the ZIP4 transporter cell surface expression and nearly absent zinc uptake, while the Val372 variant displayed significantly reduced surface protein expression, reduced basal levels of intracellular zinc, and reduced zinc uptake in comparison with the Leu372 variant. We speculate that reduced zinc uptake by the ZIP4-derived Val372 isoform may act by starving certain pathogens of zinc, and hence may have been advantageous in Sub-Saharan Africa. Moreover, these functional results may indicate differences in zinc homeostasis among modern human populations with possible relevance for disease risk.

## Author Summary

Zinc is an essential trace element with many biological functions in the body, whose concentrations are tightly regulated by different membrane transporters. Here we report an unusual case of positive natural selection for an amino acid replacement in the human intestinal zinc uptake transporter ZIP4. This substitution is recognized as one of the most strongly differentiated genome-wide polymorphisms among human populations. However, since the extreme population differentiation of this non-synonymous site was not accompanied by additional signatures of natural selection, it was unclear whether it was the result of genetic adaptation. Using computer simulations we demonstrate that such an unusual pattern can be explained by the effect of local recombination, together with positive selection in Sub-Saharan Africa. Moreover, we provide evidence to suggest functional differences between the two ZIP4 isoforms in terms of the transporter cell surface expression and zinc uptake. This result is the first genetic indication that zinc regulation may differ among modern human populations, a finding that may have implications for health research. Further, we speculate that reduced zinc uptake mediated by the derived variant may have been advantageous in Sub-Saharan Africa, possibly by reducing access of a geographically restricted pathogen to this micronutrient.

## Introduction

Zinc homeostasis is critically important for human health. Similarly to iron, zinc has manifold functions in the body, such as in the immune system [1], aging [2], DNA repair [3], signaling [4] and in diseases such as diabetes [5] and cancer [6]. On the molecular level, zinc acts as a co-factor in hundreds of metallo-enzymes as well as in hundreds of DNA-binding proteins (e.g. zinc finger proteins). Zinc homeostasis is tightly regulated by 10 zinc efflux transporters and 14 zinc influx transporters (encoded by the *SLC30A* and *SLC39A* gene families, respectively). ZIP4 (SLC39A4) is the most important intestinal zinc uptake transporter and is expressed at the apical membrane of enterocytes [7], [8]. Loss-of-function mutations in *ZIP4* cause acrodermatitis enteropathica [9], [10] [MIM 201100], a congenital disease characterized by extreme zinc deficiency if left untreated without supplemental zinc [11], [12]. Fittingly, it was recently reported that the loss of expression of this gene in a *ZIP4* intestine-specific knockout mouse caused systemic zinc deficiency, leading to disruption of the intestine stem cell niche and loss of intestine integrity [13].

The single nucleotide polymorphism (SNP) c.1114C>G (rs1871534) in the *ZIP4* gene (*SLC39A4*; NM_130849.2) results in the substitution of leucine for valine at amino acid 372 (Leu372Val) in the human ZIP4 transporter. This non-synonymous SNP is one of the most markedly differentiated genetic variants in the genome in terms of allele frequency differences between populations [14]–[16], according to data from HapMap [17], the Human Genome Diversity Panel (HGDP) [18] and the 1000 Genomes Project [16]. Extreme population differentiation is a signature of local positive selection [15], [19]–[21], but genomic scans for targets of natural selection based on other criteria, such as extended long haplotypes [22]–[24] or selective signatures in the allele frequency spectrum [25], have failed to identify *ZIP4* as a candidate gene for positive selection. To date, whether this variant has evolved under positive selection or neutrality, and its potential functional significance, has not been examined.

In the work reported here, we had three main objectives: (i) to investigate evolutionary explanations for the extreme population differentiation of the ZIP4 Leu372Val polymorphism by use of coalescent simulations; (ii) to test for functional differences in cellular zinc transport between the alleles of the Leu372Val polymorphism using a heterologous expression system; and (iii) to discuss potential selective forces behind this possibly adaptive event and their implications for zinc homeostasis in modern humans. We have extensively characterized the extreme geographical differentiation of the Leu372Val substitution and provide evidence that it has been subject to a nearly complete but mild selective sweep in Sub-Saharan Africa. Our simulations show how the extreme pattern of population differentiation, yet absence of other classical signatures of positive selection, can be explained by directional selection accompanied by the effects of a recombination hotspot near the polymorphic adaptive site. Additionally, our data demonstrate *in vitro* functional differences between the two human polymorphic alleles at codon 372 of the human ZIP4 transporter in surface protein expression, basal intracellular levels of zinc and zinc uptake. We hypothesize that the reduction in intracellular zinc levels mediated by the Val372 allele may have been advantageous in Sub-Saharan Africa, possibly by restricting access of a geographically restricted pathogen to this micronutrient, and that other possible secondary consequences for disease risk and health may result from the differential activity of the ZIP4 alleles.

## Results

### Worldwide allele frequencies

**Figure 1:**
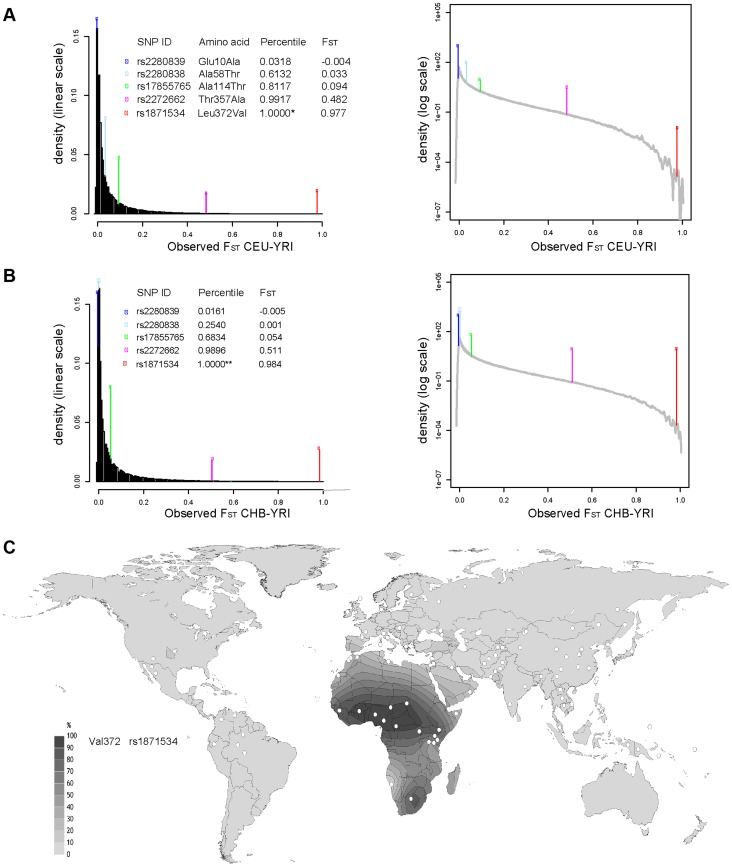
Extreme population differentiation of the Leu372Val polymorphism in *ZIP4* (*SLC39A4*). (A) Distribution of F_ST_ pairwise scores between CEU and YRI in SNPs from the 1000 Genomes data, plotted on a linear and logarithmic scale. The F_ST_ values and corresponding quantiles of the five common SNPs in ZIP4 are indicated. *The F_ST_ quantile of rs1871534 (Leu372Val) was 0.99999977. (B) Distribution of F_ST_ pairwise scores between CHB and YRI in SNPs from the 1000 Genomes data. The F_ST_ values and corresponding quantiles of the five common SNPs in ZIP4 are indicated. **The F_ST_ quantile of rs1871534 (Leu372Val) was 0.99999817. (C) Contour map of worldwide frequencies of the Val372 variant at the rs1871534 SNP. A complete list of populations and allele frequencies is available in Table S1.

Five common non-synonymous SNPs are known in the human *ZIP4* gene (Table 1): Glu10Ala (rs2280839), Ala58Thr (rs2280838), Ala114Thr (rs17855765), Thr357Ala (rs2272662) and Leu372Val (rs1871534). However, only the latter two SNPs show elevated levels of population differentiation in the 1000 Genomes Phase1 sequencing data when comparing the Yoruba from Ibadan, Nigeria (YRI) with either the Han Chinese from Beijing, China (CHB) or Utah residents of Northern and Western European origin (CEU). As shown in Figure 1A and 1B, their F_ST_ values fall above the 99.999 th percentile of the genome-wide F_ST_ distributions between CEU-YRI (with F_ST_ values for rs2272662 and rs1871534 of 0.48 and 0.98, respectively) and between CHB-YRI (with F_ST_ values of 0.51 and 0.98, respectively). We therefore verify that the Leu372Val substitution encoded by SNP rs1871534 is the non-synonymous polymorphism exhibiting the most extreme allele frequency differences in the human *ZIP4* gene. Next, we genotyped the 51 populations from the Human Genome Diversity Panel (HGDP) and compiled additional allele frequencies for this position in worldwide populations from the Alfred database [26], [27]. Additionally, we obtained new data from a Pygmy population from Gabon and North African populations of Western Sahara, Morocco, and Libya. These new data confirm that the Leu372 variant is the most common allele outside of Africa, and provide a more detailed picture of the geographical allele frequency distributions of this non-synonymous polymorphism (Figure 1C and Table S1). Overall, the Val372 variant showed the highest frequencies in Sub-Saharan Africa, with populations such as the Ibo or the Yoruban people exhibiting the most extreme derived allele frequencies worldwide (0.99 and 0.96, respectively). Interestingly, two presumably early-branching groups in Sub-Saharan Africa, the Pygmy and the San people, showed opposing trends in the derived allele frequency (0.94 and 0.0, respectively). Even though the small sample size from the San (only six individuals) means that a population frequency of up to 0.221 cannot be excluded (with p = 0.05 based on assuming Hardy-Weinberg equilibrium and a binomial approach), such divergent tendencies in these two Sub-Saharan populations are maintained. Given the elevated levels of population differentiation of the SNP rs2272662, we also genotyped the HGDP panel for the Thr357Ala polymorphism. However, compared with the Leu372Val substitution, the derived allele at this non-synonymous SNP displayed intermediate frequencies worldwide (Figure S1 and Table S1) and less extreme allele frequency differences between populations.

**Table 1:** Common non-synonymous SNPs in the *ZIP4* (*SLC39A4*) gene.

	DAF			F_ST_			Amino Acid Replacement	Functional Prediction	
SNP ID^a^	CEU	CHB	YRI	CEU-YRI	CHB-CEU	YRI-CHB		PolyPhen	SIFT
rs2280839	0.4812	0.4485	0.4545	−0.0042	−0.0030	−0.005	Glu10Ala	Benign	Deleterious
rs2280838	0.5235	0.4347	0.3803	0.0335	0.0080	0.0006	Ala58Thr	Benign	Tolerated
rs17855765	0.4812	0.4278	0.2601	0.0942	−0.0002	0.0535	Ala114Thr	Benign	Tolerated
rs2272662	0.4941	0.5401	0.0057	**0.4823**	−0.0005	**0.5110**	Thr357Ala	Benign	Tolerated
rs1871534	0.0059	0.0000	0.9787	**0.9765**	0.0002	**0.9837**	Leu372Val	PD	Deleterious

a Reported non-synonymous SNPs with minor allele frequencies (MAF) greater than 0.10 in any of the three 1000 Genomes Project populations. Values in bold are above the 99th percentile of the corresponding F_ST_ genome-wide distribution among the two compared populations. Abbreviations: DAF, Derived Allele Frequency; PD, Probably Damaging.

### Identification of Leu372 as the ancestral variant by resequencing in a Neanderthal

**Figure 2:**
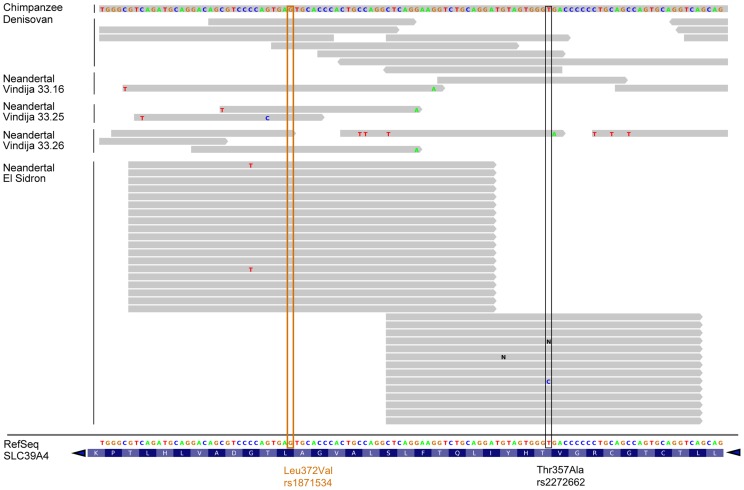
Human *ZIP4* sequence alignment with chimpanzee and archaic hominids. Archaic hominid sequences are shown from one Denisovan individual and four Neanderthal individuals (three from Vindija and one from El Sidrón, the latter resequenced for the present study). The orthologous positions of the human rs1871534 and rs227262 SNPs are shown in orange and black, respectively. Note that the reference sequence displayed at the bottom of the figure spans from position 145,639,648 to 145,639,756 in chr8 (hg19) and is the reverse complement of the *SLC39A4* coding sequence. This analysis shows that the *ZIP4* reference sequence carries the ancestral allele shared with archaic hominids and chimpanzee at these two locations.

Given the allele frequency differences observed in the Leu372Val polymorphism between the two early human branches in Africa and the uncertainty associated with the low coverage of the Neanderthal genome draft sequence [28], we resequenced the corresponding orthologous positions for rs1871534 and rs2272662 in an additional Neanderthal sample, labeled SD1253 and excavated at El Sidrón site in Spain [29]. The two positions were amplified in a multiplexed reaction, along with a diagnostic Neanderthal mitochondrial DNA (mtDNA) fragment, to monitor contamination in the PCR reaction. For the L16230-H16262 diagnostic mtDNA fragment, 64 clones were generated (Figure S2), all of which show the Neanderthal-specific 16234T-16244A-16256A-16258G haplotype [28]. This again supports the very low level of contamination in this particular sample. For the orthologous positions of the human rs1871534 and rs2272662 SNPs, 19 and 14 sequences were successfully obtained, respectively. With the exception of one clone in the second position, all sequences showed the previously inferred ancestral alleles, in agreement with the reads present for the Vindija individuals 33.16 (one read for each position), 33.25 (two for rs1871534 and none for rs2272662) and 33.26 (two and one, respectively) (Figure 2). The successful resequencing of this Neanderthal individual, together with published reads from additional Neanderthals [28] and from the Denisovan individual [30], strongly suggests that the Leu372 variant (encoded by the C allele in rs1871534) is the ancestral human form, which is also in agreement with the chimpanzee state (Figure 2). Together with the extreme population differentiation pattern, these results suggest that a selective sweep may have taken place in Sub-Saharan Africa, where the derived variant is nearly fixed.

### Extreme population differentiation explained by selection and a recombination hotspot

**Figure 3:**
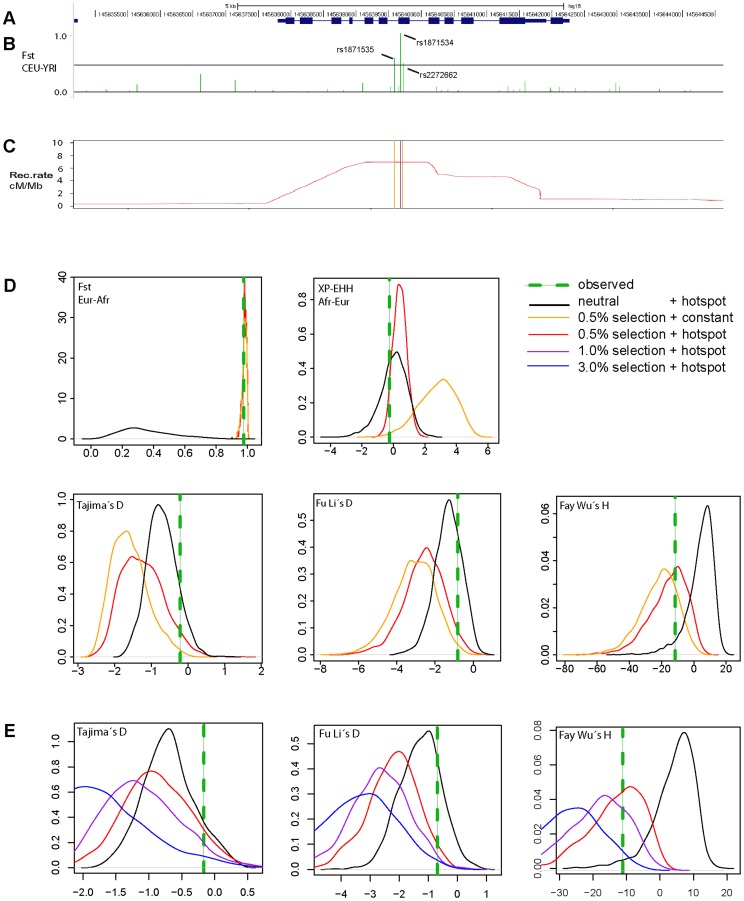
Genomic context and patterns of selection in a 10*ZIP4* (*SLC39A4*). (A) Structure of the human *ZIP4* (*SLC39A4*) gene. (B) F_ST_ scores between YRI and CEU in a 10 kb window centered in the Leu372Val polymorphism (rs1871534). The three indicated SNPs show F_ST_ scores above the 99th percentile (indicated with a black line) of the corresponding genome-wide F_ST_ distribution. (C) Recombination landscape in YRI. (D) Distributions of diverse neutrality and population differentiation statistics based on coalescent simulations [31] carried out under neutrality and different selection scenarios. The presence of a recombination hotspot of moderate strength reduced all signals of positive selection in the neutrality tests except for population differentiation. Overall, the observed values (averaged in a 10 kb window around *ZIP4*; green line) are compatible with a moderate selection coefficient (0.5%) simulated under the observed recombination landscape. (E) Coalescent simulations assuming different selection coefficients. Here, the recombination landscape was fixed to the observed landscape in YRI and we tested different selection coefficients (3%, 1%, 0.5% and 0%, the latter corresponding to neutrality). As expected, stronger selection coefficients yielded increasingly stronger deviations from neutrality.

Next we examined the complete genomic region around *ZIP4* (Figure 3) in the 1000 Genomes sequencing data. Whereas we found a cluster of three strongly elevated F_ST_ scores between CEU and YRI in the neighboring SNPs rs1871535 (intronic), rs1871534 and rs2272662 (further suggesting directional selection in a specific geographical region), in both populations there was a clear absence of extreme values in neutrality statistics such as Tajima's D or Fay and Wu's H (Figure S3). Notably, no other polymorphism in the flanking region of the human ZIP4 displays the high levels of population differentiation of the Leu372Val substitution. Interestingly, in both African and non-African populations there is a recombination hotspot in the *ZIP4* gene, which could have reduced any signature of selection on the surrounding linked variation, thereby explaining the apparent lack of significant departures from neutrality. To further investigate this possibility, we carried out coalescence simulations under a variety of recombination and selection scenarios using a well-established demography [31]. As shown in Figure 3D, the observed values for F_ST_ and most of the different neutrality statistics cannot be explained by neutral evolution or positive selection with a constant recombination rate. Instead, this atypical pattern of extreme population differentiation, yet seemingly neutral Tajima's D and other neutrality statistics, showed a higher recovery in simulations with directional selection on the derived allele in Sub-Saharan African populations in the context of the observed recombination landscape, including the hotspot (Figure 3D and 3E, Figure S4). In a more formal evaluation of the results, we quantified the empirical probability for each scenario and neutrality test as well as for different combinations of tests by using composite scores encompassing at least three complementary signatures of positive selection: (i) site frequency spectrum, (ii) population differentiation, and (iii) haplotype structure. The scenario of “weak selection (s = 0.005) + hotspot” is the most likely among the different ones tested (Table S2). Moreover, all the empirical likelihoods calculated for the different composite scores indicate that the proposed scenario of “weak selection (s = 0.005) + hotspot” is more likely than the neutral scenario (Table 2). Therefore, our simulation results indicate that the atypical patterns of selection in the *ZIP4* gene can indeed be explained by positive selection having acted upon the Val372 allele in Sub-Saharan African populations and that recombination has erased further accompanying signatures of the selective sweep. Selection coefficients lower than the ones tested (3.0%, 1.0%, 0.5%) further dilute the signal of selection in the site frequency spectrum based neutrality tests (results not shown), but require such long duration times of the sweep that would substantially predate the population split between African and Eurasian populations.

**Table 2:** Likelihood of several combinations of empirical neutrality test values around *ZIP4* in different selection scenarios versus neutrality.

Likelihood = P(Sweep)/P(Neutral)^a^			
Selection Coeficient	s = 0.005	s = 0.01	s = 0.03
All statistics combined^b^	20.105	0.524	0.003
F_ST_ - XPEHH - Tajima's D	18.518	3.321	0.271
F_ST_ - XPEHH - Fu Li's D	5.925	0.498	0.025
F_ST_ - XPEHH - Fay Wu's H	326.386	41.330	1.393

a Likelihoods were computed from the combined empirical probabilities obtained when considering the observed recombination landscape in *ZIP4* (see Table S2)

b Neutrality statistics include F_ST_ for population differentiation; XPEHH for extended linkage disequilibrium decay; and Tajima's D, Fu Li's D and Fay Wu's H for site frequency spectrum.

### Functional effect of Leu372Val

**Figure 4:**
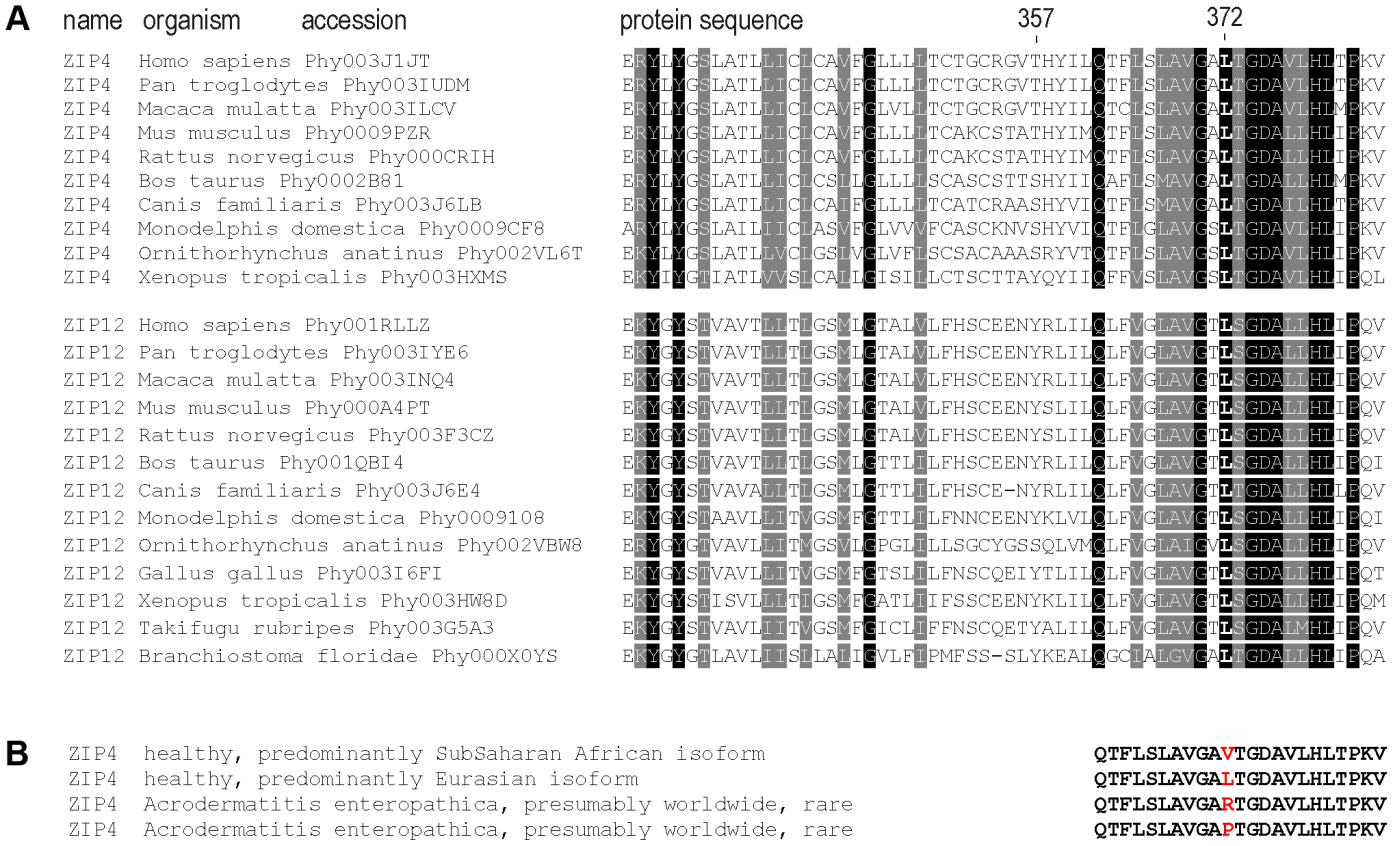
Sequence conservation and clinical relevant variation around the 372 ZIP4 position. (A) Sequence conservation across the vertebrate species tree and the sister protein families SLC39A4 (ZIP4) and SLC39A12 (ZIP12). The highly conserved position Leu372 and the less conserved position Thr357 are indicated. Sequences were downloaded from PhylomeDB [72] and aligned in T-Coffee [73]. (B) Human amino acid variation around the 372 position in acrodermatitis patients and healthy individuals.

We observed that the Leu372Val polymorphism affects a highly conserved amino acid (Figure 4) and that the same codon position has been altered in acrodermatitis patients carrying missense mutations Leu372Arg [32] and Leu372Pro [8]. Moreover, both PolyPhen [33] and SIFT [34] algorithms predict functional effects for the Leu372Val substitution (see Table 1). These observations led us to test the Leu372Val polymorphism for a possible functional change in the ZIP4 transporter, using transiently transfected HeLa cells. To be able to control for possible haplotypic effects between the two most highly differentiated non-synonymous SNPs in the ZIP4 transporter, we also considered variation at the Thr357Ala polymorphism in the functional analyses. Furthermore, we introduced the pathological mutations Leu372Arg and Leu372Pro in the Ala357 background of the human *ZIP4* gene and analyzed them as well. The pathological impact of the Leu372Pro mutation on ZIP4 protein biology and function has already been evaluated in the mouse ZIP4 protein [10], but not the Leu372Arg mutation. Besides providing confirmation of their impact in the context of the human gene, the use of these pathological mutations provided us with an extreme phenotype to which to compare the phenotype associated with the *ZIP4* non-synonymous polymorphisms. In all cases, functional analyses were carried out to determine effects on expression, subcellular localization, and zinc transport.

**Figure 5:**
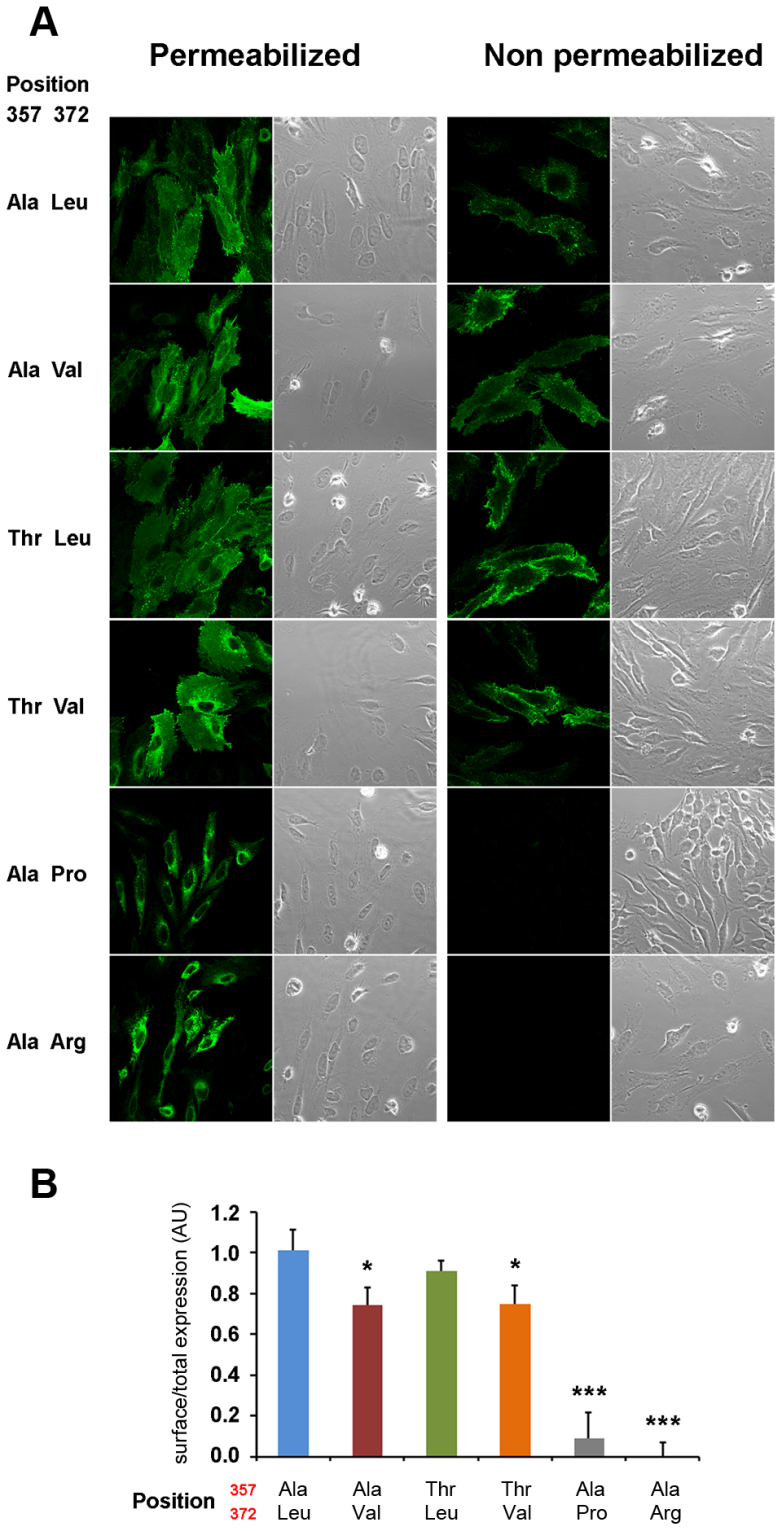
Val372 shows reduced membrane surface expression. (A) Immunostaining of the different isoforms of ZIP4 in HeLa cells under permeabilizing (left) conditions for total protein visualization and non-permeabilizing (right) conditions for surface protein visualization. Bright field images are provided to show the presence of cells on the field in all conditions. The acrodermatitis enteropathica variants Leu372Pro and Leu372Arg show absence of membrane expression. (B) Surface expression quantification normalized by the total amount of transporter of the different ZIP4 isoforms obtained from 12 independent measurements obtained in 4 different transfections. Data are expressed as mean ± SEM. * P<0.05 and *** P<0.001 *vs* Ala357-Leu372, one way ANOVA. The two isoforms expressing Val372 show reduced surface expression compared to the Leu372 isoforms.

As shown in Figure 5, human ZIP4 proteins carrying the Leu372Pro and Leu372Arg mutations showed an absence of surface protein expression (P<0.001, one way ANOVA versus the Ala357-Leu372 isoform), consistent with the known causal role of these variants in the zinc deficiency disorder, acrodermatitis enteropathica. Interestingly, the derived Val372 variant also showed significantly decreased surface expression, but to a much lesser extent, and independently of the Thr357Ala substitution (P<0.05 in both Ala357 and Thr357 backgrounds; one way ANOVA versus the Ala357-Leu372 isoform). Overall, the Leu372Val substitution had a highly significant effect on surface expression (ANOVA, p = 0.00021), while there was no effect ascribable to the Thr357Ala replacement (p = 0.579). Western blot analysis of all isoforms revealed a remarkable decrease in detection of the Ala357-Pro372 isoform (Figure S5A). However, the reduced expression of this isoform was not due to a defect in the construct sequence but to a higher protein degradation rate, as shown in Figure S5B. Further analysis showed that the Ala357-Leu372 and Ala357-Val372 isoforms do not differ in protein degradation rate. Therefore, the differences in the surface expression experiment must be due to a different trafficking pattern of these variants. In this sense, co-localization of ZIP4 with calnexin (a protein present in the lumen of the endoplasmic reticulum) indeed showed that those proteins presenting lower surface expression were partially retained in the endoplasmic reticulum (Figure S6).

**Figure 6:**
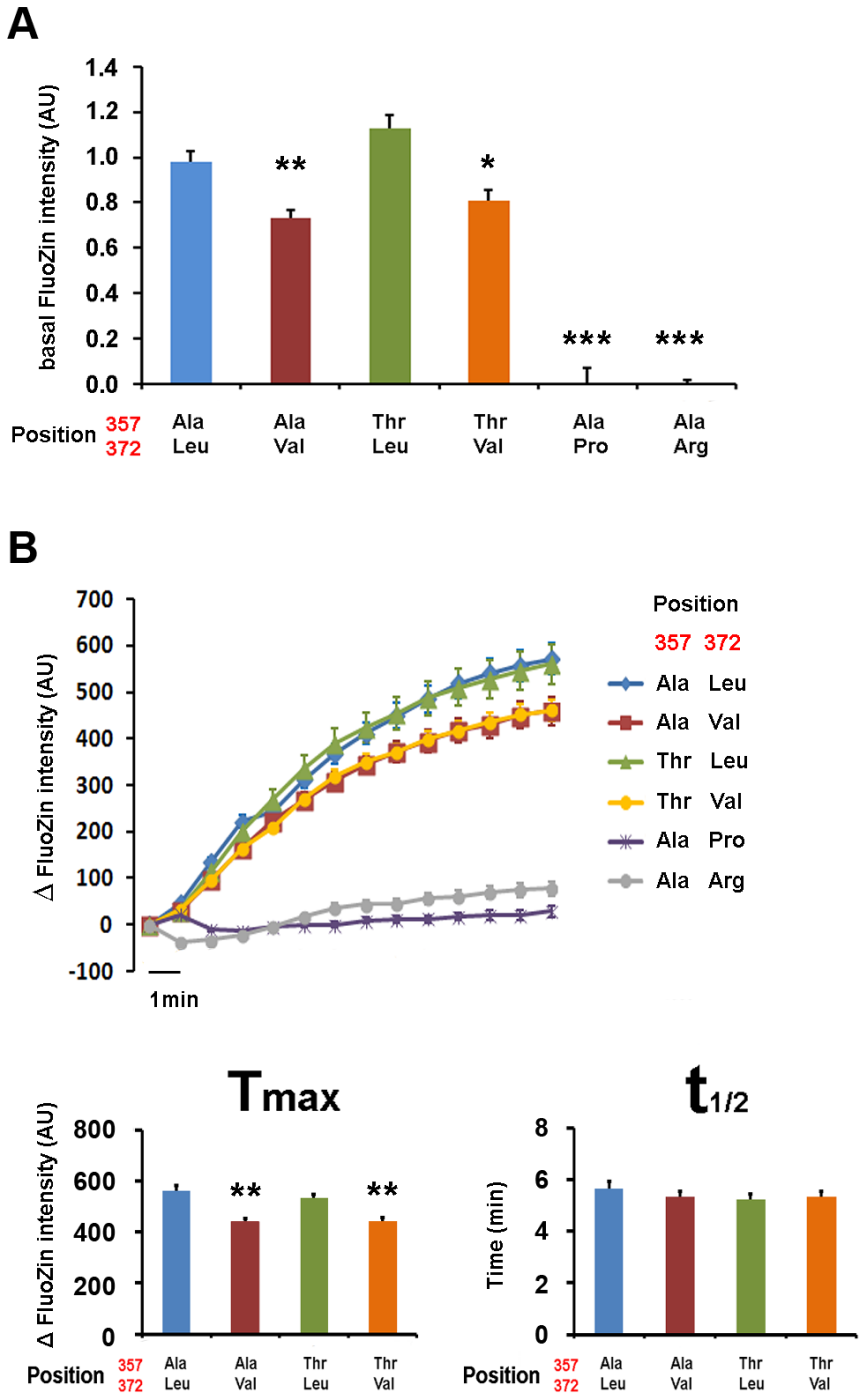
Val372 shows reduced zinc uptake transport. (A) Basal zinc content in HeLa cells transiently transfected with different ZIP4 isoforms plus empty CFP vector. Transfected cells were compared with surrounding non-transfected cells. The two isoforms expressing Val372 show significantly reduced intracellular zinc. (B) Zinc uptake upon perfusion with 200 µM ZnSO4 external solution. Graph bars show the maximum transport (Tmax) and the time to reach half of Tmax (t1/2) for the different isoforms that reach the plasma membrane, following the color code on the left. Data are presented as mean ± SEM of 3 different transfections and more than 25 cells per condition. Significance was calculated using ANOVA, with the Ala357-Leu372 isoform as reference (*p<0.05, **p<0.01, ***p<0.001). The two isoforms expressing Val372 show reduced Tmax but no difference in t1/2 when compared to the Leu372 isoforms.

Zinc transport analysis of the different ZIP4 isoforms was performed in two ways. First, we quantified basal zinc content with FluoZin-3 in HeLa cells overexpressing the various ZIP4 variants during a 24-hour period (Figure 6A), and second, we recorded intracellular zinc uptake upon perfusion with an external solution containing 200 µM Zn^2+^ (Figure 6B). Our results show that basal zinc content in cells overexpressing pathological variants Pro372 and Arg372 did not differ from surrounding non-transfected HeLa cells. On the contrary, all common ZIP4 variants (Ala357, Thr357, Leu372 and Val372) promoted increased intracellular zinc levels. However, and in agreement with their reduced surface expression, Val372 variants (in both Ala357 and Thr357 backgrounds) presented lower basal zinc content compared to Leu372 (P<0.01 and P<0.05, respectively; one way ANOVA versus the Ala357-Leu372 isoform; Figure 6A). As shown in Figure 6B, cells overexpressing the pathological Leu372Arg and Leu372Pro mutations did not uptake zinc, consistent with their inability to traffic to the plasma membrane. Zinc uptake mediated by the Val372 variants was also consistent with their reduced membrane expression; i.e. the Val372 variants in both Ala357 and Thr357 backgrounds presented significantly lower maximum transport (T_max_) compared to the Leu372 variant (P<0.01 in each case; Figure 6B). However, the time to reach half-maximal transport (t_1/2_) showed no significant difference, indicating that transport kinetics were not markedly different among the four common variants (Figure 6). Overall, these results support the idea that the Val372 variant does not disturb the kinetics of the ZIP4 transporter but leads to lower zinc uptake transport due to reduced surface expression.

## Discussion

### Leu372Val as the target of an atypical selective sweep in Africa

Our study was triggered by the observation of extreme population differentiation between Sub-Saharan African and non-African populations involving the Leu372Val polymorphism in the *ZIP4* gene, unaccompanied by any other signals of a classic hard sweep, such as long extended haplotype homozygosity, in either population (Figures S3, S7 and S8). By interrogating and compiling allele frequencies in more than 100 worldwide human populations, we further characterized the extreme population differentiation of the Leu372Val polymorphism and confirmed that this result was not an artifact of allele switching [15]. Given the worldwide distribution of the human derived and ancestral alleles (confirmed by sequencing a Neanderthal and phylogenetic conservation), we conclude that this sweep must have taken place within Africa, probably in Sub-Saharan Africa, and not outside the African continent. Notably, the extreme population differentiation of the Leu372Val polymorphism represents the top fourth region within the global genome-wide F_ST_ distribution between CEU-YRI obtained from the 1000 Genomes Project data. The only CEU-YRI F_ST_ values that are more extreme all involve well-known examples of local geographical adaptation in humans: the *SLC24A5* and *SLC45A2* genes (with an F_ST_ of 0.9826 and 0.9765, respectively), which have been associated with light skin pigmentation in Europeans; and the *DUFFY* gene (with an F_ST_ of 0.9765), which provides resistance to the malaria pathogen *Plasmodium vivax*. Moreover, with the notable exception of *DUFFY* FY*O allele [35], [36], most of the extreme F_ST_ values obtained when comparing Africans with non-Africans are usually attributed to local adaptation outside of Africa. Our detection of such a rare signature of natural selection in the African continent is therefore quite remarkable. Interestingly, it is congruent with a recent study that has found only limited evidence for classical sweeps in African populations, which is likely due to a combination of limitations of the currently used methodology and specific characteristics of African population history [37].

Notably, we observed a nearly complete but mild selective sweep for the Val372 variant in Africa, which involves three SNPs with extremely high population differentiation, whereas most other commonly used tests for selection show values not even close to genome-wide significance. Our coalescent simulations indicate that this unusual pattern might be explained by local positive selection in combination with an observed recombination hotspot of moderate strength. At approximately 7 cM/Mb, the recombination rate is only around 7-fold higher than the genomic background, but the hotspot is extended over 3–4 kb. Therefore, a similar number of recombination events may accumulate over time corresponding to a more typically sized hotspot of 1 kb and a recombination rate of around 25 cM/Mb. To our knowledge, this is the first example of a nearly complete selective sweep that is obscured by the effect of a recombination hotspot. It is compatible with earlier theoretical observations that instances of weaker selection in the presence of recombination may not always have an influence on polymorphism statistics [38] and with the observed effect of recombination on the partial sweep around the malaria-related β-globin gene [39]. Because of the unclear effects of the recombination hotspot, it was not possible to estimate the age of the sweep using linkage disequilibrium decay related methods (e.g. [40]). It is likely that a mild selection pressure would have needed a long time to reach the extreme population differentiation values observed, indicating this may be an ancient event. The fact that the high frequency of the Val372 allele is restricted to Sub-Saharan African populations suggests that the selection process started after the Out of Africa expansion of modern humans (i.e. sixty thousand years ago). Alternatively, it is also possible that the bottleneck in the Out of Africa expansion did not sample the Val372 allele, which in turn could explain its absence in most non-African populations. This implies that the Out of Africa event is not a hard upper limit for the age of the selection process.

Other more complex evolutionary scenarios cannot be entirely ruled out, and could warrant a more detailed investigation. For example: (i) selection acting on standing genetic variation, in the sense that the Val372 variant was already segregating when it came under the influence of local selection; (ii) additional directional selection against the Val372 allele in non-African populations; (iii) selection favoring the Leu372 variant on multiple, geographically independent origins mostly in non-African populations, in addition to positive selection on the Val372 variant in Africa; and (iv) ‘gene surfing’ of any of the two variants on the wave of a population range expansion [41]. However, we consider it is unnecessary to invoke such complex scenarios in preference to the simpler one we propose based on coalescent simulations. Moreover, back-and-forth migrations between Sub-Saharan African, Northern African and Middle Eastern populations after the first Out-of-Africa wave of migration [42] could easily explain the observed low-intermediate allele frequencies in Middle Eastern populations without invoking additional selection events.

In the absence of additional linked functional variants in the region, we infer that directional selection has acted on the *ZIP4* gene. This conclusion is supported by: (i) the disease phenotype of acrodermatitis enteropathica, which involves extreme and potentially lethal zinc deficiency and is caused by, among others, diverse mutations at amino acid position 372 in ZIP4 [43]; (ii) the absence of cellular zinc transport in Leu372Arg and Leu372Pro acrodermatitis mutants; (iii) the finding that the Val372 variant leads to reduced zinc transport at the cellular level; and finally (iv) the conservation of this amino acid position across diverse species (Figure 4). Furthermore, we infer that the Leu372Val substitution was the functional site targeted by selection due to its location in the predicted center of selection (highest F_ST_), and since it is the only putative functional polymorphism in the *ZIP4* gene. Of the other two polymorphic variants with somewhat high allele frequency differences between populations, the Thr357Ala substitution (rs2272662) does not show any functional effect and the intronic rs1871535 cannot be associated with any known regulatory function (according to information on DNAse I hypersensitivity clusters, CpG Islands and transcription factor binding sites available from the ENCODE data (http://genome.ucsc.edu/ENCODE [44]). Therefore, both rs1871535 and rs2272662 are likely to be neutral. Other non-synonymous polymorphisms with intermediate allele frequencies in the *ZIP4* gene (Glu10Ala, Ala58Thr, and Ala114Thr) have very low F_ST_ scores and are therefore not considered candidate variants for selection.

### Possible consequences at the cellular and organ level

Our functional results in transfected HeLa cells indicate that the Val372 form of the ZIP4 receptor has lower relative cell surface expression, despite no expected differences in mRNA expression and protein synthesis. Interestingly, we found that this decreased expression translated into reduced zinc transport of the derived Val372 variant at the cellular level. That is, we observed differences in the maximal transport (T_max_) with no significant differences in the transport kinetics (T_1/2_) between Leu372 and Val372. The functional results observed in transfected HeLa cells are likely to be transferable to other epithelial cells, in accordance with independent experiments showing an effect of acrodermatitis variants at position 372 on surface expression (in CHO cells) and on zinc transport (in HEK293 cells) when using mouse cDNA [10]. However, the critical function of ZIP4 in knockout studies has been shown to primarily affect intestinal zinc uptake [13].

In contrast to the Leu372Pro and Leu372Arg acrodermatitis mutations, which served as controls and showed an almost complete absence of zinc transport, both the Leu372 and Val372 variants must be capable of carrying out zinc transport in the normal range of concentrations, given their high frequency in the healthy population. The consequences of this difference in zinc transport at the organ and organismal level are currently unclear, although there is a strong indication that this variant may indeed be phenotypically relevant. For example, a similar non-synonymous mutation in the porcine homologue of ZIP4 leads to non-pathogenic reduced tissue concentrations of zinc in piglets [45].

### Nutritional immunity as a putative selective force

Could the concept of “nutritional immunity” [46], [47] involving zinc explain a putative selective force in Sub-Saharan Africa? According to this hypothesis, the human host restricts access to certain micronutrients, so that pathogens become less virulent. This is a well-known mechanism of immune defense mediated by iron metabolism [48], and there are indications that zinc metabolism could have a similar function [47], [49]. For example, hypoferremia and hypozincemia are both part of the acute phase response to infection and both seem to be influenced by a different zinc transporter from the same family, ZIP14 [50]. We speculate that the selective force behind the extreme F_ST_ pattern of the Leu372Val substitution may be related to pathogens or infectious diseases. It is known that decreased zinc uptake mediated by ZIP4 leads to decreased zinc concentrations in the major organs, as shown in a mouse knockout model [13]. While the phenotypic effect of the Val372 allele in humans is currently unknown, we conjecture that the *in vitro* difference may indeed translate into physiological differences, possibly leading to a slightly decreased uptake of dietary zinc. Fittingly, there is suggestive evidence that African genetic ancestry may involve lower serum levels of zinc [51], as African-American children have a fourfold risk of zinc deficiency compared to Hispanic children. This result would suggest that African ancestry may be associated with lower serum zinc levels, although these results may be biased due to differences in lifestyle, socio-economic status etc., and this observation would need to be confirmed by controlled studies. Alternatively, lower zinc concentrations mediated by the Leu372Val substitution in the enterocyte cells could facilitate early diarrheal episodes during a digestive infection in order to reduce the pathogen load on the luminal surface [52], [53]. Similarly, the lower level of expression of the ZIP4 isoform carrying the Val372 variant could also be advantageous if any parasite uses the ZIP4 receptor to enter enterocytes. Furthermore, the selective force may be related to pre-historic differences in dietary zinc due to lifestyle or to local levels of zinc concentrations in soil and the food chain.

### Potential implications – towards a phenotype

No large-scale ethnic comparisons related to serum or tissue zinc concentrations are available. To our knowledge, rs1871534 has not been tested in case-control studies in African populations related to one of the numerous existing infectious diseases like malaria, trypanosmias or Lhassa fever. It is therefore possible that important evidence for a possible selective force has been missed. In future research, the inclusion of additional cell lines, and genotype-phenotype association studies in diverse ethnic populations may help to clarify further phenotypic consequences of this non-synonymous polymorphism. Genotype-phenotype association studies should involve African-American or East African populations in which the Val372 allele is segregating at intermediate frequencies. Candidate phenotypes and traits to interrogate could be serum zinc concentrations, zinc content in hair and nails, serum zinc concentrations after controlled zinc supplementation, and a range of disease traits, especially diseases with an elevated risk in different populations, for example, diverse types of cancer in African Americans. As this SNP was not included in the commonly used Affymetrix and Illumina SNP arrays with up to one million variants (although it is included in several of the latest arrays), potential clinically relevant associations may have been missed. Interestingly, common polymorphisms in other zinc transporters show genome-wide associations with disease traits, such as a non-synonymous variant in the zinc efflux transporter ZnT8 (SLC30A8) and diabetes incidence [54], as well as a regulatory variant in the zinc influx transporter ZIP6 (SLC39A6) and survival in esophagal cancer [55].

### Conclusions

The identification of a high-frequency derived allele polymorphism in the *ZIP4* zinc transporter gene (*SLC39A4*), combined with a more complete picture of worldwide allele frequencies and in-depth coalescent simulations, is consistent with a long lasting selective event in Sub-Saharan Africa driven by a moderate selection coefficient. This event did not leave the typical footprint of a selective sweep with long haplotypes or detectable neutral deviations in the allele frequency spectrum of the surrounding region, most likely because of the presence of a moderate recombination hotspot. Through functional experiments we have verified the Leu372Val substitution as the likely causal site. Given that two functionally different alleles of this key component of cellular zinc uptake are distributed so divergently across worldwide populations, our results may point to functional differences in zinc homeostasis among modern human populations with possible broader relevance for health and disease.

## Materials and Methods

### Samples and genotyping

The G and C alleles at rs1871534 (Leu372Val) have been swapped in various public sources such as HapMap (http://www.hapmap.org) or dbSNP (http://www.ncbi.nlm.nih.gov/SNP) that report conflicting allele frequencies in populations with a similar geographical origin. This situation led us to repeat the genotyping of this SNP in the Human Genome Diversity Panel (HGDP-CEPH) [18]. We also genotyped rs2272662 (which causes the Thr357Ala substitution) because, within the *ZIP4* gene, it shows the second highest allele frequency differences between CEU and YRI HapMap populations and allele frequencies were not available at the worldwide level. The rs1871534 and rs2272662 loci were genotyped in the H971 subset [56] of the HGDP-CEPH [18], representing 51 worldwide populations, and in an additional population from Africa: Pygmies from Gabon (N = 39)[57]. We also genotyped rs1871534 in North African populations from Western Sahara (Saharawi, N = 50), Morocco (Casablanca, N = 30; Rabat, N = 30; Nador, N = 30) and Libya (Libyans, N = 50). Genotyping was performed using Taqman assays C__11446716_10 and C__26034235_10 on an Applied Biosystems Light Cycler (7900HR), according to standard protocols. Additional genotypes for rs1871534 were obtained from the Alfred database (http://alfred.med.yale.edu) [26], [27].

### Ethics statement

Informed consent was obtained for all human samples analysed and genotyping analyses were performed anonymously. The project obtained the ethics approval from the Institutional Review Board of the local institution (Comitè Ètic d'Investigació Clínica - Institut Municipal d'Assistència Sanitària (CEIC-IMAS) in Barcelona, Spain.

### Neanderthal resequencing

The El Sidrón Neanderthal sample SD1253 has been used in many paleogenomic studies due to its high endogenous DNA content and low contamination levels [28], [58]–[62], attributable in part to having been extracted using an anti-contamination protocol [63]. In addition, it has the advantage of having been dated to 49,000 years ago [64], prior to the arrival of modern humans to Europe. The two orthologous positions for rs1871534 and rs2272662 were amplified using a two-step PCR protocol [59] in a multiplexed reaction along with a diagnostic Neanderthal mitochondrial DNA (mtDNA) fragment. After visualizing the PCR products in a low-melting temperature agarose gel, the bands were excised, purified and cloned using the TOPO-TA cloning kit (Invitrogen). Inserts of the correct size were sequenced on an ABI3730 XL capillary sequencer (Applied Biosystems).

### Simulations

Simultaneous coalescent simulation of recombination hotspots and selection were carried out using Cosi v1.2 [31], [65]. As the underlying neutral demography, we used the best-fit model of Shaffner et al. [31], [65] with slight modifications (Table S3), similar to a previously used approach [66]. In particular, the migration frequencies were set to zero and the time points of the European and African population bottlenecks were moved back to 3,300 generations before present to accommodate the long sweep times resulting from the lowest selection coefficient we used (0.5%). The sweep was shifted back 350 generations to retain the final population expansions with the advantage of (i) a better approximation to the fitted model, and (ii) the generation of sufficient singletons when compared to the 1000 Genomes Phase1 data. Subsequent thinning of the simulated data was performed by removing 48% of singleton positions across all populations to account for the underestimation of singletons in 1000 Genomes data. This correction step yielded a much improved (although not perfect) unfolded site frequency spectrum as displayed by the derived allele frequencies (DAF) and a F_ST_ distribution that closely matched the empirical data from 1000 genomes (Figure S9). Specifically, we compared the empirical F_ST_ and DAF distributions from the 1000 genomes data against the original demographic “best-fit” model [31] and two models adapted to allow for different selective sweeps (the one from [66] and that applied in the current study). As seen in Figure S9, our modified model matched the empirical data as well as or better than the other demographic models.

For each subsequent simulation, we used either the recombination landscape including hotspots from the YRI population provided by the 1000 Genomes Consortium and based on HapMap 2 trio data (http://1000genomes.org) or alternatively a constant recombination rate of 8.17×10^−9^, which was calculated as the mean recombination rate in the 100 kb window surrounding *ZIP4*. Simulations had a length of 100 kb, were run in 500 replicates for each scenario and sample sizes were set to 176 chromosomes for Sub-Saharan Africans and 194 chromosomes for Europeans. Regions under positive selection were modeled using a single causal variant that rose to an allele frequency of 0.98 corresponding approximately to that observed today in YRI. We simulated three different selection coefficients (0.5%, 2% and 3%) that led to different durations of the sweep: 2,938 generations (∼60,000–85,000 years for generation times of 20 and 29 years, respectively;[67]), 1,469 generations (∼30,000–43,000 years), or 458 generations (∼10,000–13,000 years).

Empirical probabilities and likelihoods for the different selection statistic values observed in *ZIP4* were estimated under each simulated selection scenario (see Table 2). Firstly, the empirical percentile in which each observation was found was estimated for each test (F_ST_, dDAF, Tajima's D, Fay and Wu's H, Fu Li's D and XP-EHH) and scenario (neutral + constant recombination, neutral + hotspot recombination, low selection + constant recombination, medium selection + constant recombination, high selection + constant recombination, low selection + hotspot recombination, medium selection + hotspot recombination, high selection + hotspot recombination). This percentile was then subtracted from one if it was higher than 0.5 and multiplied by two to mimic a two-tailed test. Thus, if the observed value was found at the median of the simulated distribution, it yielded a probability of one. By contrast, if it was found in a tail of the distribution, it yielded a probability close to zero. For each scenario, we computed the combined empirical probability for several set combinations of observed neutrality test values by multiplying each corresponding empirical probability (Table S2). Each combination contained at least one neutrality statistic capturing each of the three main signatures of selection explored (population differentiation, haplotype structure or the site frequency spectrum). Next, empirical likelihoods were estimated as the ratio of the combined empirical probability under each selection scenario over the same probability under neutrality only for the hotspot recombination landscape observed in *ZIP4* (Table 2). Likelihoods for the different combinations of statistics containing dDAF in Table S2 were nearly identical to the equivalent combinations obtained with F_ST_ (data not shown). As a conservative decision given the high correlation between F_ST_ and dDAF, we do not present the likelihood of any combination including both statistics. It is important to point out that any of the currently available human demographies in combination with coalescent simulators have relatively severe limitations mainly (i) in terms of the number of included populations (e.g. African populations) (ii) the accuracy and timing of the demographic events and (iii) the option to include selective sweeps as well as a defined recombination landscape. Therefore it is clear that the complexities of possible evolutionary scenarios (as discussed in the main text) are beyond what can be modeled by current approaches.

### Neutrality tests on simulated and the 1000 Genomes data

Neutrality tests on simulated and the 1000 Genomes population data were performed as described by Pybus et al. [68] and using the 1000 Genomes Selection Browser (http://hsb.upf.edu). Briefly, Tajima's D, Fu and Li's D and Fay and Wu's H were calculated using a sliding window approach with 30 kb windows and approximately 3 kb offset. F_ST_ [69] and XP-EHH [70] between CEU and YRI were calculated for each polymorphic position.

### Cells and reagents

Human *ZIP4* cDNA encoding the long isoform of the protein and the Ala357 and Leu372 variants was cloned into pcDNA 3.1 (+) expression vector together with a hemagglutinin (HA) tag at the carboxyl terminus as described previously [71]. The Leu372Pro and Leu372Arg mutants, as well as the Thr357Ala and Leu372Val polymorphisms, were introduced via site-directed mutagenesis following standard conditions (QuikChange II XL; Stratagene; see Table S4 for complete human cDNA and primers used in the mutagenesis). The six human *ZIP4* isoforms obtained (i.e. Ala357-Leu372, Ala357-Val372, Thr357-Leu372, Thr357-Val372 as well as Ala357-Pro372 and Ala357-Arg372) were confirmed by sequencing with the ABiPrism 3.1 BigDye kit before their use in transfection experiments. HeLa cells were cultured in DMEM plus 10% FBS and, subsequently, each of the various *ZIP4* forms were transiently transfected using polyethyleneimine as the transfection reagent (PolySciences).

### Immunodetection

For the cell surface expression experiments, live cells were incubated with anti HA (1∶1000) in DMEM without serum for 1h at 37° before fixation with 4% paraformaldehyde. After blocking for 30 min (1% BSA, 2% FBS in PBS), cells were incubated with a secondary antibody (1∶2000) for 45 min in the blocking solution. For the total cell expression experiments, cells were permeabilized with 0.1% Triton in PBS for 10 min after fixation. Following blocking for 30 min (1% BSA, 2% FBS in PBS), cells were incubated in the blocking solution with anti HA (1∶1000) for 1 h 30 min, washed with PBS and incubated with the secondary antibody (1∶2000) for 45 min. Images were acquired using an inverted Leica SP2 confocal microscope with a 40×1.32 Oil Ph3 CS objective. Expression was quantified by measuring chemiluminescence with a plate reader (24-well plates) using peroxidase-linked anti-mouse antibody (GE Healthcare) as a secondary antibody and SuperSignal West Femto reagent as a substrate (Thermo scientific). Data are presented as the ratio between surface expression and total expression of the transporter. Statistical significance was tested using standard ANOVA.

### Zinc measurements

Cells were transiently transfected with the various *ZIP4* isoforms plus empty ECFP vector for 24–36 h. Cytosolic Zn2+ signal was determined in CFP-positive cells loaded with FluoZin3 2.5 µM (Invitrogen) in a solution containing 140 mM NaCl, 5 mM KCl, 1.2 mM CaCl_2_, 0.5 mM MgCl_2_, 5 mM glucose, 10 mM HEPES, 300 mosmol/l, pH 7.4 for 20 min. Cytosolic [Zn2+] increases are presented as the difference with respect to the basal signal of emitted fluorescence (510 nm) after adding 200 µM ZnSO4 in a continuous perfusion bath. The kinetics of the various isoforms were calculated using a sigmoidal non-linear regression. In the same set of experiments, basal cellular Zn2+ content was estimated as the difference in FluoZin intensity between transfected cells and non-transfected cells before adding Zn2+ to the bath. Flourescence intensity was measured using an Olympus IX70 inverted fluorescence microscope, controlled by Aquacosmos software (Hamamatsu).

## Supporting Information

Figure S1 Worldwide allele frequencies for the Leu372Val (rs1871534, top) and Thr357Ala (rs2272662, bottom) polymorphisms. Circles are not proportional to sample sizes. Maps were generated with MapViewer. Complete list of population and sample sizes analyzed are given in Table S1.
(TIF)

Figure S2 Neanderthal mt-DNA control for contamination.
(TIF)

Figure S3 Patterns of selection in a genomic region of 100 kb around the *ZIP4* (*SLC39A4*) gene for three human populations. Gene context and summary of tests for positive selection obtained from the 1000 Genomes data for three populations: Yoruba from Ibadan, Nigeria (YRI), Han Chinese from Beijing, China (CHB) and Utah residents with Northern and Western European origin (CEU). With the exception of population differentiation (here: the F_ST_ statistic), those statistics based on site frequency (Tajima's D, Fu and Li's D, Fay and Wu's H) and haplotype structure (XPEHH) do not reach genome-wide significance (not shown) in any of the three populations.
(TIF)

Figure S4 Patterns of selection in a genomic region of 100 kb around the *ZIP4* gene (*SLC39A4*). (A). Gene context and summary of tests for positive selection obtained in the Yoruba population from the 1000 Genomes data. Those statistics based on the site frequency spectrum (Fay and Wu's H, Fu and Li's D and Tajima's D) show weakly negative scores near *ZIP4* that do not approach genome-wide significance (not shown), so they should not be regarded as indicative of positive selection. Those statistics based on population differentiation (here: F_ST_) show three SNPs (see Figure 1) with elevated values between CEU and YRI. One of them, rs1871534 (Leu372Val), is among the most highly differentiated SNPs in the genome. (B) Fine-scale recombination rate from the Yoruba population plotted on a linear scale reveals a moderate recombination hotspot near *SLC39A4*. (C) Detailed view of simulated values along the 100 kb region for different statistical tests of positive selection assuming different scenarios comparable to Figure 1: (i) no selection and considering the observed recombination landscape from the Yoruba population (black lines); (ii) a selective sweep in the West African population and a constant recombination rate (orange lines); and (iii) a selective sweep in the West African population and the observed recombination landscape including the hotspot (red lines). Statistics were calculated in a sliding window approach with 30 kb windows and approximately 3 kb offset. For F_ST_ only the maximum score for each window was considered. Solid lines indicate median values and dashed lines indicate the 5th and the 95th percentiles of 500 replicated simulations.
(TIF)

Figure S5 Detection of ZIP4 isoforms by western blot. (A) Gel was loaded with 80 µg of total protein extracts from HeLa cells transiently transfected with the different ZIP4 isoforms. Anti-HA antibody (1∶1000) was used to detect the transporters and anti-beta actin (1∶3000) as a loading control. (B) HeLa cells transfected with the Ala357-Leu372, Ala357-Val372, and Ala357-Pro372 isoforms were treated with 10 µg/ml cyclohexamide for different time periods (1 h, 3 h, 6 h and 8 h). Total protein extracts were obtained and western blotting was performed. A representative experiment for each isoform is shown (left). The quantification analysis normalized the band intensity to the initial amount of protein before the treatment (time 0) (right). This experiment was performed three times per isoform (n = 3).
(TIF)

Figure S6 Retention of ZIP4 in the endoplasmatic reticulum. Immunostaining under permeabilizing conditions on cells expressing different ZIP4 variants using anti-HA (1∶1000) for ZIP4 detection and anti-calnexin (1∶1000) (Abcam) as an endogenous endoplasmic reticulum maker protein.
(TIF)

Figure S7 Linkage disequilibrium plot for the YRI population in a 50 kb window around the *ZIP4* (*SLC39A4*) gene. The plot was generated with Haploview and using HapMap 2 data (release 21).
(TIF)

Figure S8 Haplotype visualization in a 40 kb window around the *ZIP4* (*SLC39A4*) gene. Plots from the HapMap browser (http://hapmap.ncbi.nlm.nih.gov) are shown for the Yoruba, Han Chinese and French populations. There is no indication of extended haplotype patterns that could indicate a classical selective sweep in any of the three populations.
(TIF)

Figure S9 Demographic models versus empirical data. Empirical results based on the 1000 genomes data (only chromosome 1) are compared to an established demographic model [31] and against two demographic models adapted for capturing selective sweeps (Grossman et al. [66] and the present study) using neutral simulations of 500 kb length (500 replicates; roughly matching the length of chromosome 1). (A) Derived allele frequency distributions based on data or simulations reflecting African and European genetic origin. (B) F_ST_ distributions in a pair-wise population approach. As indicated in the text, the thinning of random singleton positions improves the fit of simulated data based on site frequency spectra.
(TIF)

Table S1 Worldwide allele frequencies for the Leu372Val (rs1871534) and Thr357Ala (rs2272662) polymorphisms.
(PDF)

Table S2 Empirical probabilities under neutrality and different selection scenarios.
(PDF)

Table S3 Simulation parameters similar to best-fit model Schaffner et al. [31].
(PDF)

Table S4 Description of primers and hcDNA used in mutagenesis.
(PDF)
