# Supplementary figures and images for: Extreme Population Differences in the Human Zinc Transporter ZIP4 (SLC39A4) Are Explained by Positive Selection in Sub-Saharan Africa
Source: PLoS Genet. 2014 Feb 20;10(2):e1004128. doi: 10.1371/journal.pgen.1004128 (PMC3930504; doi:10.1371/journal.pgen.1004128)

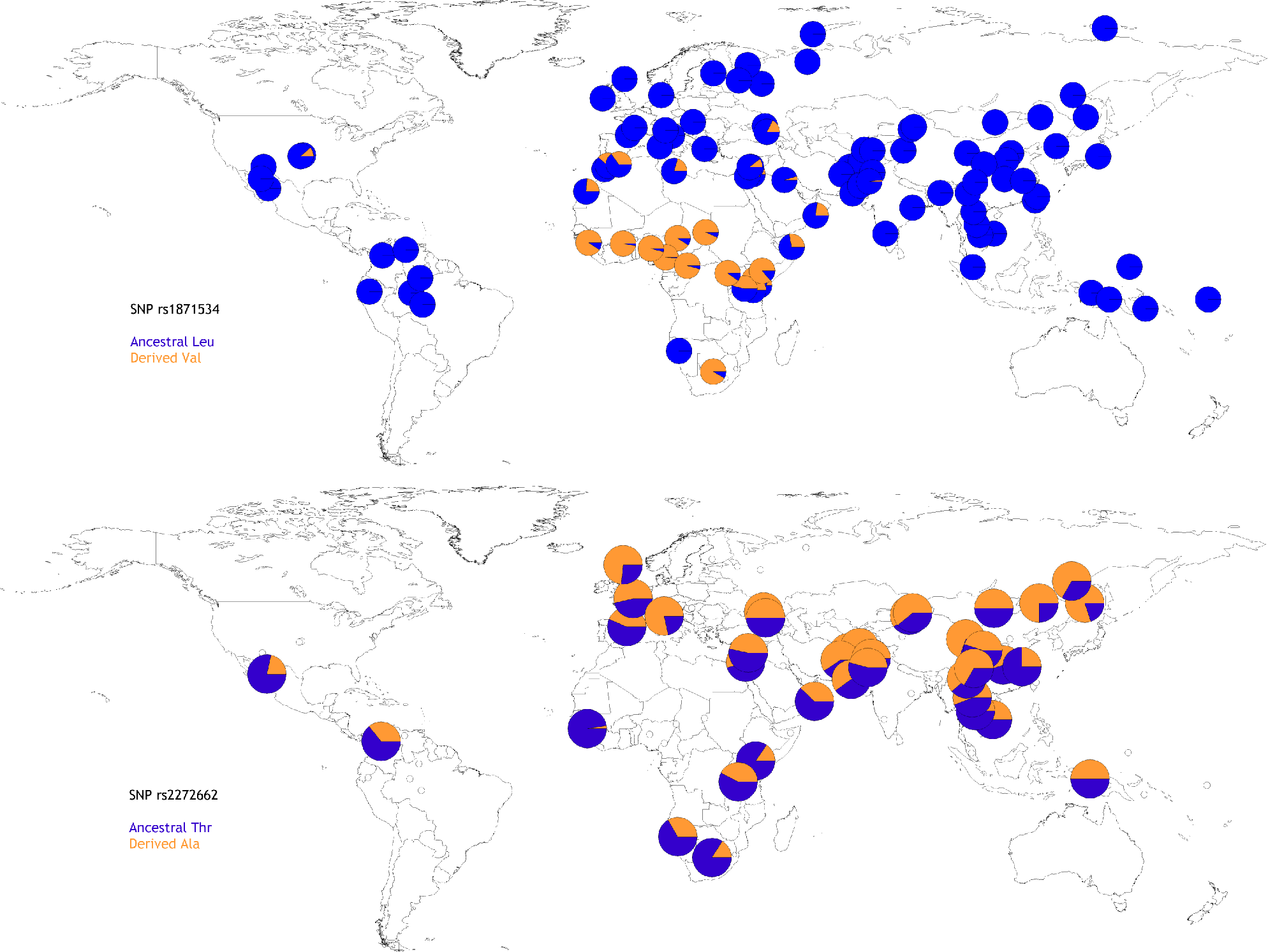

Supplement: Figure S1 — Worldwide allele frequencies for the Leu372Val (rs1871534, top) and Thr357Ala (rs2272662, bottom) polymorphisms. Circles are not proportional to sample sizes. Maps were generated with MapViewer. Complete list of population and sample sizes analyzed are given in Table S1. (TIF) [file pgen.1004128.s001.tif]

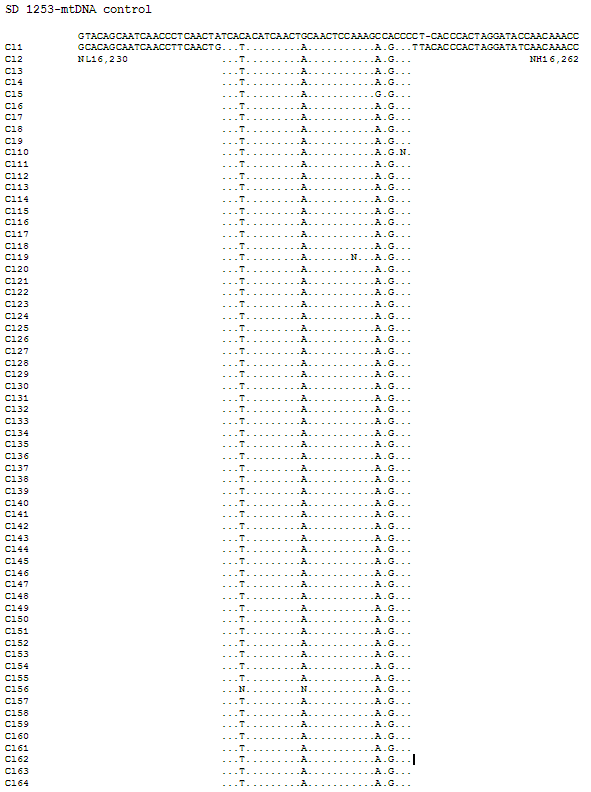

Supplement: Figure S2 — Neanderthal mt-DNA control for contamination. (TIF) [file pgen.1004128.s002.tif]

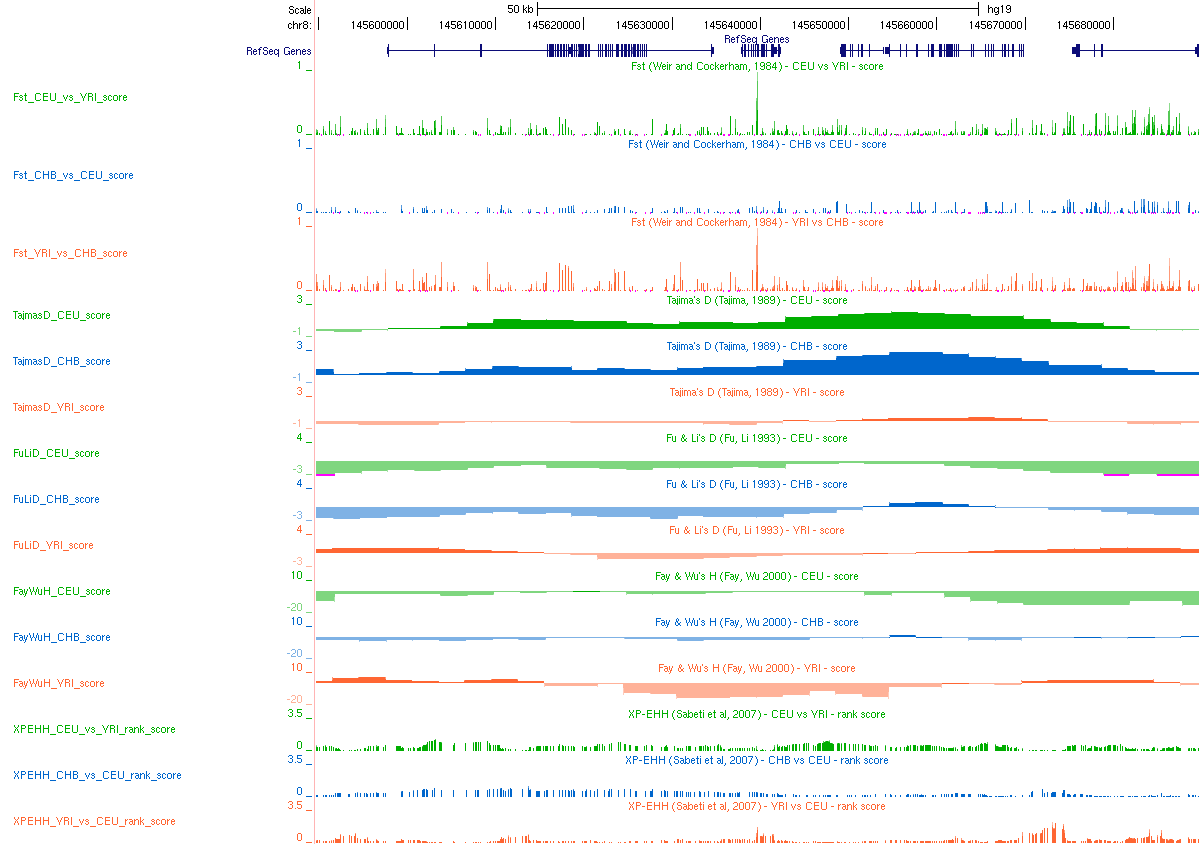

Supplement: Figure S3 — Patterns of selection in a genomic region of 100 kb around the ZIP4 (SLC39A4) gene for three human populations. Gene context and summary of tests for positive selection obtained from the 1000 Genomes data for three populations: Yoruba from Ibadan, Nigeria (YRI), Han Chinese from Beijing, China (CHB) and Utah residents with Northern and Western European origin (CEU). With the exception of population differentiation (here: the FST statistic), those statistics based on site frequency (Tajima's D, Fu and Li's D, Fay and Wu's H) and haplotype structure (XPEHH) do not reach genome-wide significance (not shown) in any of the three populations. (TIF) [file pgen.1004128.s003.tif]

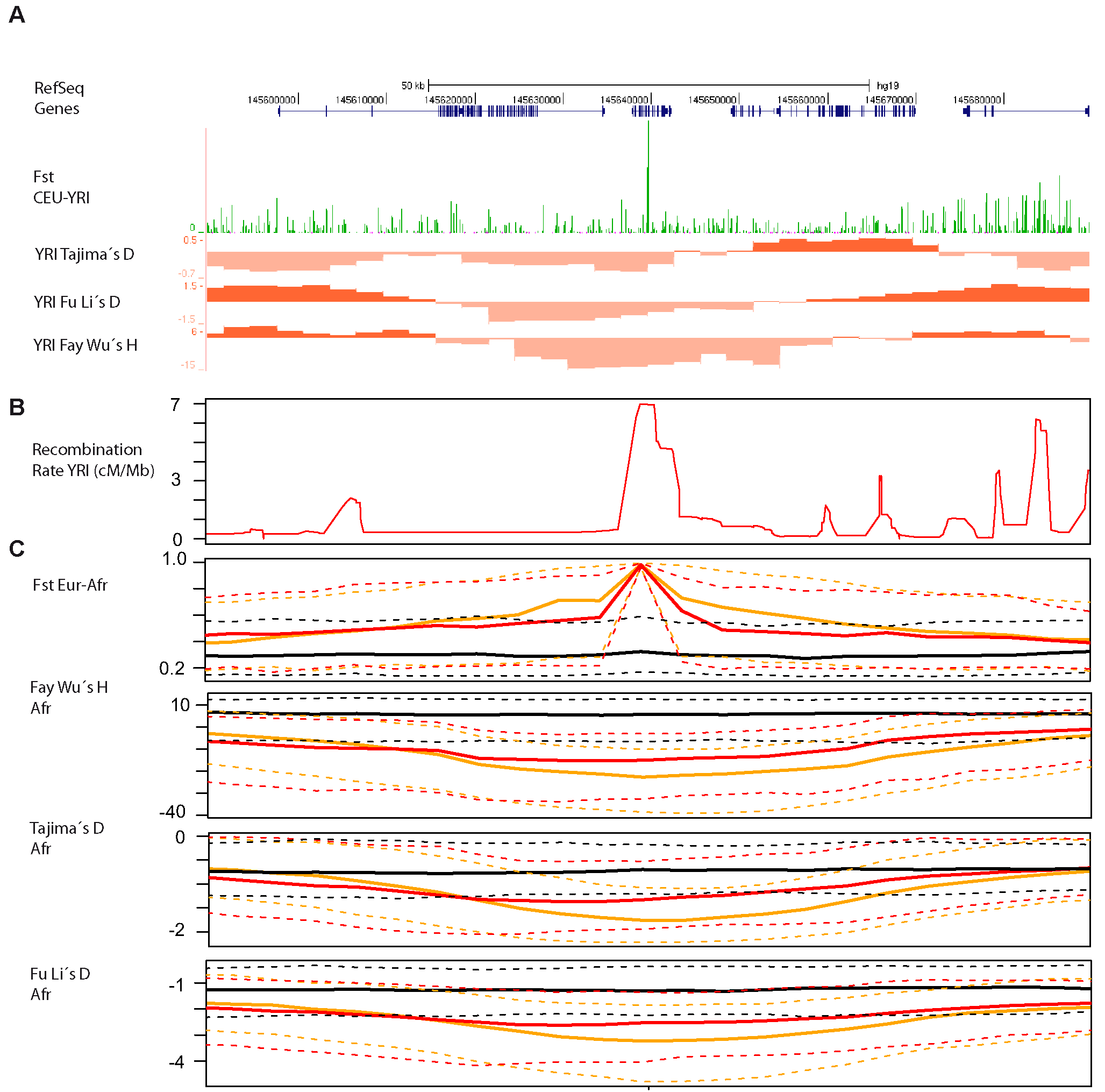

Supplement: Figure S4 — Patterns of selection in a genomic region of 100 kb around the ZIP4 gene (SLC39A4). (A). Gene context and summary of tests for positive selection obtained in the Yoruba population from the 1000 Genomes data. Those statistics based on the site frequency spectrum (Fay and Wu's H, Fu and Li's D and Tajima's D) show weakly negative scores near ZIP4 that do not approach genome-wide significance (not shown), so they should not be regarded as indicative of positive selection. Those statistics based on population differentiation (here: FST) show three SNPs (see Figure 1) with elevated values between CEU and YRI. One of them, rs1871534 (Leu372Val), is among the most highly differentiated SNPs in the genome. (B) Fine-scale recombination rate from the Yoruba population plotted on a linear scale reveals a moderate recombination hotspot near SLC39A4. (C) Detailed view of simulated values along the 100 kb region for different statistical tests of positive selection assuming different scenarios comparable to Figure 1: (i) no selection and considering the observed recombination landscape from the Yoruba population (black lines); (ii) a selective sweep in the West African population and a constant recombination rate (orange lines); and (iii) a selective sweep in the West African population and the observed recombination landscape including the hotspot (red lines). Statistics were calculated in a sliding window approach with 30 kb windows and approximately 3 kb offset. For FST only the maximum score for each window was considered. Solid lines indicate median values and dashed lines indicate the 5th and the 95th percentiles of 500 replicated simulations. (TIF) [file pgen.1004128.s004.tif]

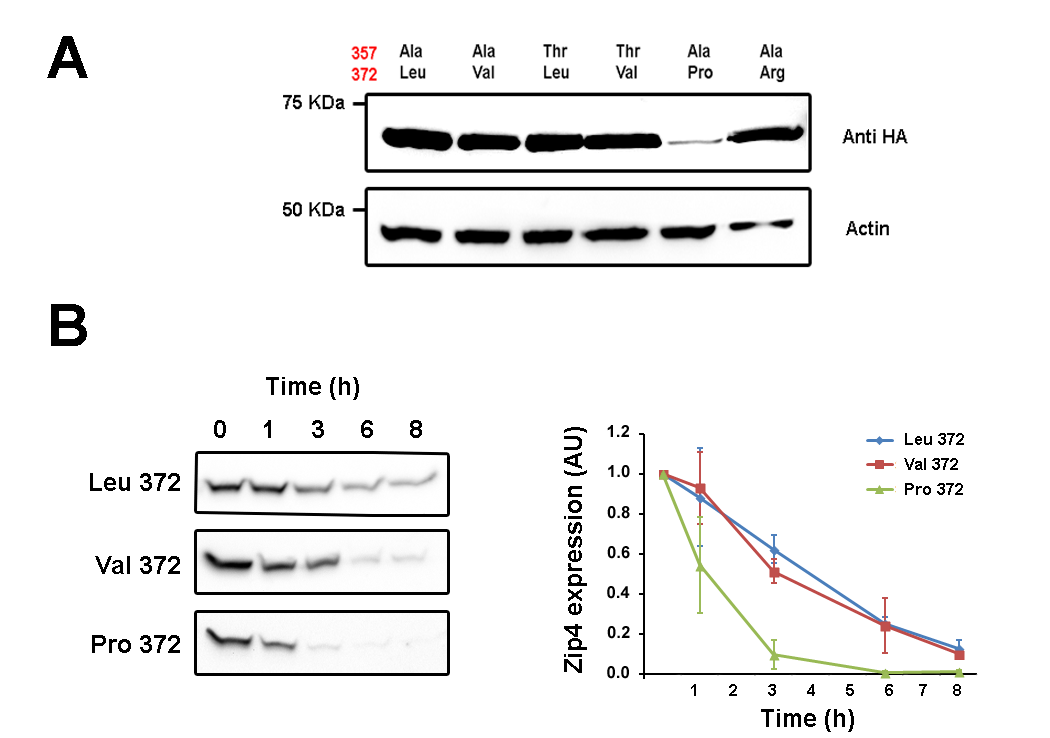

Supplement: Figure S5 — Detection of ZIP4 isoforms by western blot. (A) Gel was loaded with 80 µg of total protein extracts from HeLa cells transiently transfected with the different ZIP4 isoforms. Anti-HA antibody (1∶1000) was used to detect the transporters and anti-beta actin (1∶3000) as a loading control. (B) HeLa cells transfected with the Ala357-Leu372, Ala357-Val372, and Ala357-Pro372 isoforms were treated with 10 µg/ml cyclohexamide for different time periods (1 h, 3 h, 6 h and 8 h). Total protein extracts were obtained and western blotting was performed. A representative experiment for each isoform is shown (left). The quantification analysis normalized the band intensity to the initial amount of protein before the treatment (time 0) (right). This experiment was performed three times per isoform (n = 3). (TIF) [file pgen.1004128.s005.tif]

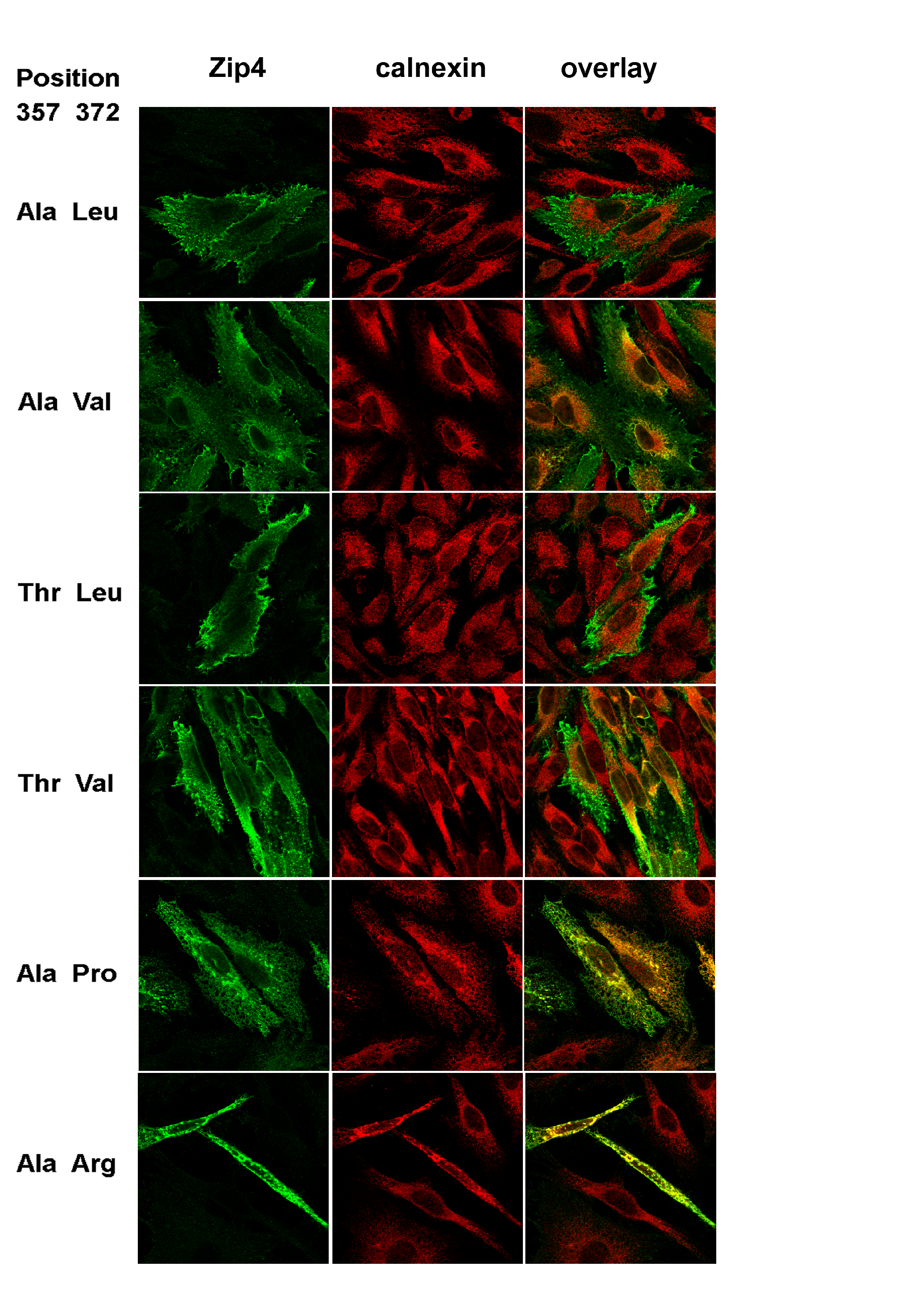

Supplement: Figure S6 — Retention of ZIP4 in the endoplasmatic reticulum. Immunostaining under permeabilizing conditions on cells expressing different ZIP4 variants using anti-HA (1∶1000) for ZIP4 detection and anti-calnexin (1∶1000) (Abcam) as an endogenous endoplasmic reticulum maker protein. (TIF) [file pgen.1004128.s006.tif]

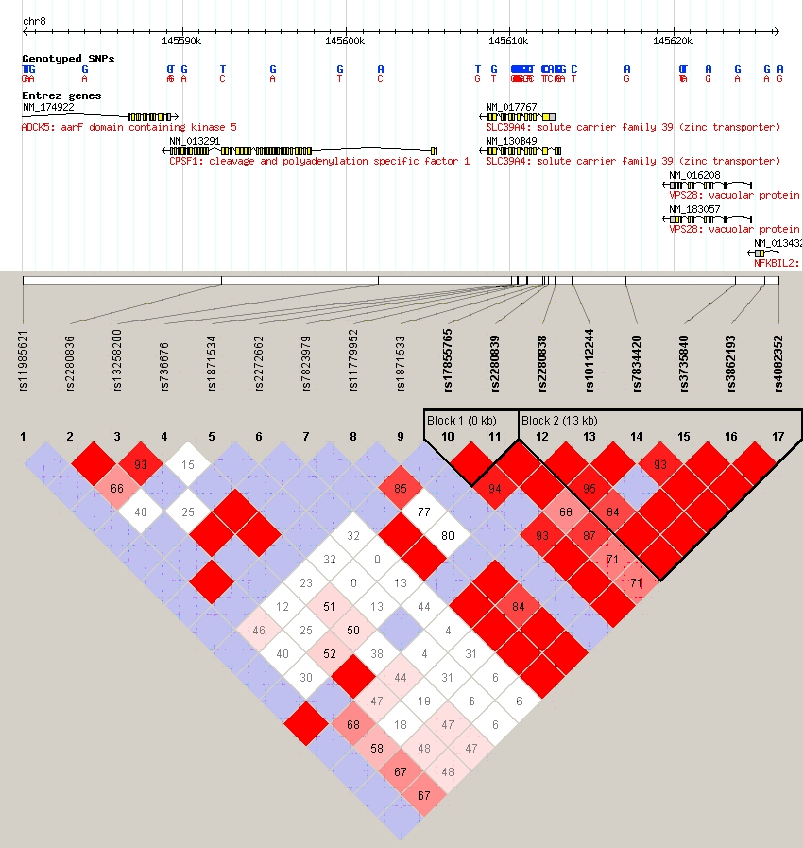

Supplement: Figure S7 — Linkage disequilibrium plot for the YRI population in a 50 kb window around the ZIP4 (SLC39A4) gene. The plot was generated with Haploview and using HapMap 2 data (release 21). (TIF) [file pgen.1004128.s007.tif]

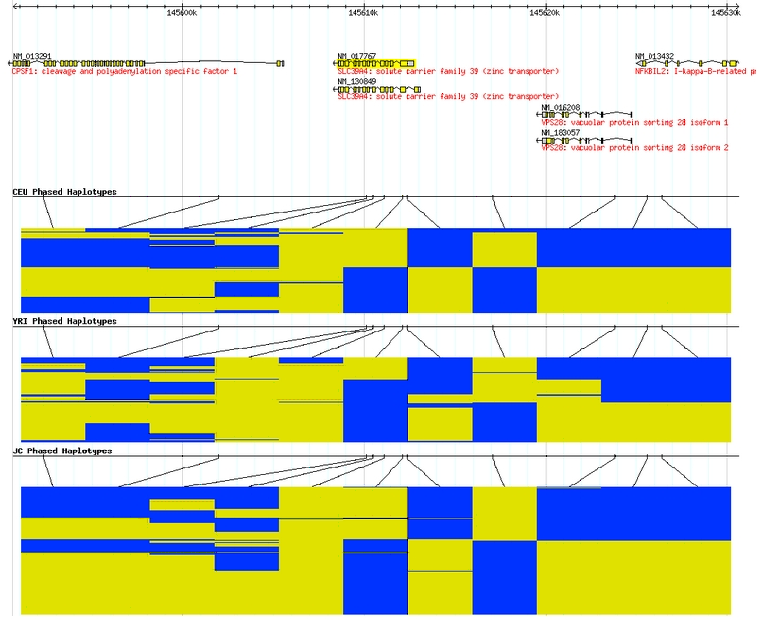

Supplement: Figure S8 — Haplotype visualization in a 40 kb window around the ZIP4 (SLC39A4) gene. Plots from the HapMap browser (http://hapmap.ncbi.nlm.nih.gov) are shown for the Yoruba, Han Chinese and French populations. There is no indication of extended haplotype patterns that could indicate a classical selective sweep in any of the three populations. (TIF) [file pgen.1004128.s008.tif]

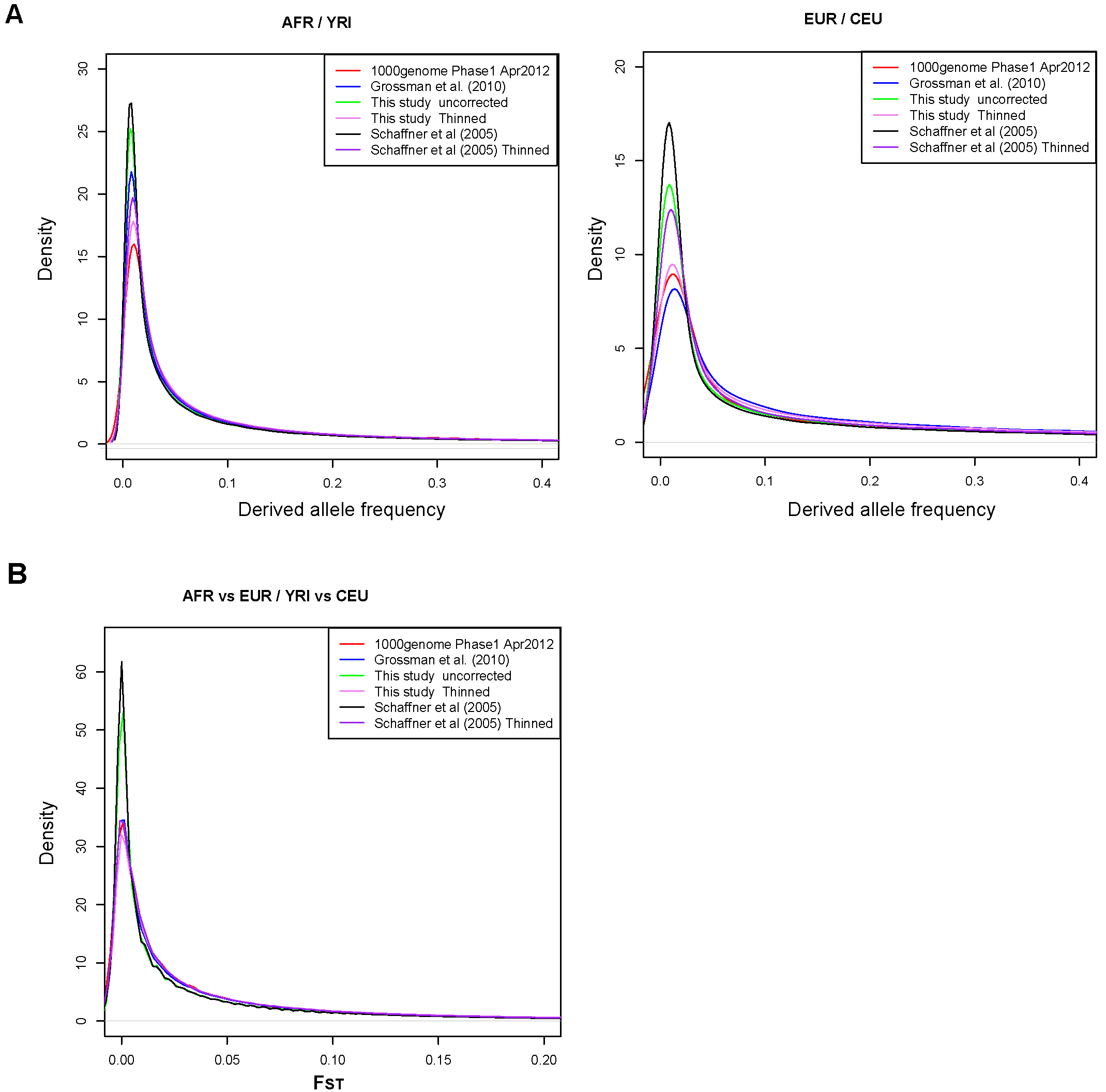

Supplement: Figure S9 — Demographic models versus empirical data. Empirical results based on the 1000 genomes data (only chromosome 1) are compared to an established demographic model [31] and against two demographic models adapted for capturing selective sweeps (Grossman et al. [66] and the present study) using neutral simulations of 500 kb length (500 replicates; roughly matching the length of chromosome 1). (A) Derived allele frequency distributions based on data or simulations reflecting African and European genetic origin. (B) FST distributions in a pair-wise population approach. As indicated in the text, the thinning of random singleton positions improves the fit of simulated data based on site frequency spectra. (TIF) [file pgen.1004128.s009.tif]
