# Supplementary material for: Extreme Population Differences in the Human Zinc Transporter ZIP4 (SLC39A4) Are Explained by Positive Selection in Sub-Saharan Africa
Source: PLoS Genet. 2014 Feb 20;10(2):e1004128. doi: 10.1371/journal.pgen.1004128 (PMC3930504; doi:10.1371/journal.pgen.1004128)
Supplement: Table S1 — Worldwide allele frequencies for the Leu372Val (rs1871534) and Thr357Ala (rs2272662) polymorphisms. (PDF) [file pgen.1004128.s010.pdf]

Table S1. Worldwide allele frequencies for the Leu372Val (rs1871534) and Thr357Ala (rs2272662) polymorphisms

Order	Population	Origin	Geographic Coordinates	Source	rs1871534 2N	rs1871534 Val Allele	rs2272662 2N	rs2272662 Ala Allele
1	Morocco (Casablanca)	North Africa	33.53N, 7.58W	Present study	52	0.327		
2	Morocco (Rabat)	North Africa	34N, 6.85W	Present study	18	0.389		
3	Morocco (Nador)	North Africa	35.16N, 2.93W	Present study	20	0.350		
4	Libyans	North Africa	32.88N, 13.16E	Present study	92	0.196		
5	Saharawi	North Africa	25N, 13W	Present study	58	0.241		
6	African Americans	Africa	25-65N, 65-125W	Alfred	174	0.701		
7	Bantu	Africa	29S, 30E	HGDP	36	0.917	38	0.158
8	Chagga	Africa	2.5-3.5S, 37-38E	Alfred	88	0.750		
9	Hausa	Africa	7-18N, 4-20E	Alfred	76	0.908		
10	Ibo	Africa	5-7N, 5-10E	Alfred	94	0.989		
11	Lisongo	Africa	4-11.5N, 14-27E	Alfred	14	0.929		
12	Luhya	Africa	0.6N , 34.8E	HapMap	92	0.859	92	0.152
13	Maasai	Africa	0N, 37.9E	HapMap	90	0.456	92	0.424
14	Mandenka	Africa	12N, 12W	HGDP	42	0.905	44	0.023
15	Pygmy (Biaka)	Africa	4N,17E	Alfred	134	0.955		
16	Pygmy (Gabon)	Africa	2.13N, 12.05E	Present study	78	0.974	70	0.100
17	Pygmy (Mbuti)	Africa	1N, 29E	Alfred	74	0.892		
18	San	Africa	21S, 20E	HGDP	12	0.000	12	0.333
19	Sandawe	Africa	4-7S, 35-38E	Alfred	78	0.462		
20	Somali	Africa	12N-2S, 40-52E	Alfred	32	0.281		
21	Yoruba	Africa	6-10N, 2-8E	Alfred	148	0.959		
22	Zaramo	Africa	4-11S, 36-40E	Alfred	66	0.864		
23	Ami	Asia	22.5-24N, 121-121.5E	Alfred	78	0.000		
24	Atayal	Asia	21.75-25.5N, 120.5-122.5E	Alfred	82	0.000		
25	Balochi	Asia	30-31N, 66-67E	HGDP	48	0.042	48	0.458
26	Brahui	Asia	30-31N, 66-67E	HGDP	48	0.021	48	0.479
27	Burusho	Asia	36-37N, 73-75E	HGDP	50	0.000	48	0.521
29	Cambodian	Asia	10.5-14.5N, 102.5-107.5E	Alfred	44	0.000		
30	Dai	Asia	21N, 100E	HGDP	20	0.000	20	0.300
31	Daur	Asia	48-49N, 124E	HGDP	20	0.000	20	0.750
32	Druze	Asia	32.5-34N, 35-37E	Alfred	198	0.056		
33	Hakka	Asia	22-35N, 105-122E	Alfred	80	0.000		
34	Han	Asia	22-40N, 100-120E	Alfred	114	0.000		
35	Hazara	Asia	24-38N, 56-73E	Alfred	194	0.005		
36	Hezhen	Asia	47-48N, 132-135E	HGDP	20	0.000	20	0.800
37	Japanese	Asia	30-46N, 130-146E	Alfred	94	0.000		
38	Kachari	Asia	27-27.5N, 94-95.5E	Alfred	26	0.000		
39	Kalash	Asia	35-37N, 71-72E	HGDP	46	0.000	42	0.595
40	Keralite	Asia	8-13N, 75-77.5E	Alfred	54	0.000		

41	Khanty	Asia	59-67N, 65-88E	Alfred	98	0.000		
42	Komi-Zyrian	Asia	59-69N, 46-66E	Alfred	90	0.000		
43	Koreans	Asia	34.5-43N, 124.5-130.5E	Alfred	106	0.000		
44	Kuwaiti	Asia	28-30N, 46-49E	Alfred	22	0.045		
45	Lahu	Asia	22N, 100E	HGDP	16	0.000	16	0.563
46	Lao Loum	Asia	14-23N, 100-107.5E	Alfred	224	0.000		
47	Makrani	Asia	26N, 62-66E	HGDP	50	0.020	50	0.600
48	Malaysians	Asia	1-7N, 100-119E	Alfred	20	0.000		
49	Miaozu	Asia	28N, 109E	HGDP	18	0.000	20	0.550
50	Mohanna	Asia	23-27N, 66-68E	Alfred	96	0.000		
51	Mongola	Asia	48-49N, 118-120E	HGDP	20	0.000	20	0.500
52	Naxi	Asia	26N, 100E	HGDP	18	0.000	18	0.611
53	Negroid Makrani	Asia	23-27N, 61-68E	Alfred	48	0.167		
54	Oroqen	Asia	48-53N, 122-131E	HGDP	18	0.000	18	0.667
55	Pashtun	Asia	24-39N, 61-77E	Alfred	192	0.000		
56	Pathan	Asia	32-35N, 69-72E	HGDP	50	0.000	50	0.620
57	She	Asia	27N, 119E	HGDP	20	0.000	20	0.250
58	Sindhi	Asia	24-27N, 68-70E	HGDP	48	0.021	48	0.458
59	Thoti	Asia	13-20N, 77-84E	Alfred	24	0.000		
60	Tu	Asia	36N, 101E	HGDP	20	0.000	20	0.700
61	Tujia	Asia	29N, 109E	HGDP	20	0.000	20	0.450
62	Uygur	Asia	44N, 81E	HGDP	20	0.000	20	0.600
63	Xibo	Asia	43-44N, 81-82E	HGDP	18	0.000	18	0.611
64	Yakut	Asia	55-74N, 105-165E	Alfred	100	0.000		
65	Yizu	Asia	28N, 103E	HGDP	20	0.000	18	0.667
66	Adygei	Europe	45-44N, 39-40.5E	Alfred	106	0.000		
67	Basque	Europe	43N, 0	HGDP	48	0.000	46	0.435
68	Chuvash	Europe	54.5-56.5N, 46-48.5E	Alfred	82	0.000		
69	Danes	Europe	54.7-58N, 8-13E	Alfred	100	0.000		
70	Europeans (Mixed)	Europe	35-70N, 24W-56E	Alfred	176	0.000		
71	Finns	Europe	60-75N, 20-35E	Alfred	66	0.000		
72	French	Europe	46N, 2E	HGDP	56	0.000	54	0.537
73	Greeks	Europe	35-41.6N, 19.5-28.5E	Alfred	100	0.000		
74	Hungarian	Europe	45.5-48.5N, 16-23E	Alfred	170	0.000		
75	Irish	Europe	51-56N, 6-11W	Alfred	224	0.000		
76	Italian	Europe	37.9-47N, 7-18.5E	Alfred	178	0.006		
77	Orcadian	Europe	59N, 3W	HGDP	30	0.000	30	0.733
78	Russians	Europe	45-85N, 30-180E	Alfred	92	0.000		
79	Samaritans	Europe	31.75-32.25N, 34.5-35.5E	Alfred	76	0.000		
80	Sardinian	Europe	38.75-41.25N, 8-10E	Alfred	66	0.000		
81	Tuscan	Europe	40N, 9E	HGDP	16	0.000	14	0.786
82	Adygei	Middle East	44N, 39E	HGDP	34	0.000	34	0.794
83	Bedouin	Middle East	31N, 35E	HGDP	92	0.174	90	0.500
84	Druze	Middle East	32N, 35E	HGDP	80	0.025	84	0.548
85	Jews (Ashkenazi)	Middle East		Alfred	226	0.018		
86	Jews (Ethiopian)	Middle East	12-15N, 35-40E	Alfred	72	0.208		

87	Jews (Sephardic)	Middle East		Alfred	48	0.083		
88	Jews (Yemenite)	Middle East	12-18N, 43-53E,	Alfred	80	0.050		
89	Mozabite	North Africa	32N, 3E	HGDP	56	0.232	58	0.379
90	Palestinian	Middle East	32N, 35E	HGDP	90	0.100	92	0.467
91	Cheyenne	North America	34-37N, 97-101W	Alfred	112	0.000		
92	Maya	North America	18-20N, 90-88W	Alfred	96	0.052		
93	Mvskoke	North America	34.6-36.2N, 95.2-96.7W	Alfred	18	0.110		
94	Pima	North America	29N, 108W	HGDP	28	0.000	28	0.214
95	Pima (Arizona)	North America	33-34N, 111-112.5W	Alfred	102	0.000		
96	Pima (Mexico)	North America	28-30.5N, 108-109.5W	Alfred	104	0.000		
97	Melanesian	Oceania	5-7S, 154-156E	Alfred	44	0.000		
98	Micronesians	Oceania	0-15S, 135-165E	Alfred	66	0.000		
99	Papuan	Oceania	4S, 143E	HGDP	30	0.000	30	0.500
100	Papuan New Guinean	Oceania	0.5-11.5S, 130.5-154E	Alfred	42	0.000		
101	Samoans	Oceania	13-14.5S, 169-173W	Alfred	16	0.000		
102	Colombian	South America	3N, 68W	HGDP	14	0.000	14	0.357
103	Guihiba	South America	5-6.5N, 67-72W	Alfred	22	0.000		
104	Karitiana	South America	9-9.5S, 63.5-65W	Alfred	110	0.000		
105	Quechua	South America	13-14S, 71-73W	Alfred	42	0.000		
106	Surui	South America	9-11.5S, 60-62W	Alfred	84	0.000		
107	Ticuna	South America	2.5-4.5S, 67-71W	Alfred	128	0.000		
