# Supplementary material for: Extreme Population Differences in the Human Zinc Transporter ZIP4 (SLC39A4) Are Explained by Positive Selection in Sub-Saharan Africa
Source: PLoS Genet. 2014 Feb 20;10(2):e1004128. doi: 10.1371/journal.pgen.1004128 (PMC3930504; doi:10.1371/journal.pgen.1004128)
Supplement: Table S2 — Empirical probabilities under neutrality and different selection scenarios. (PDF) [file pgen.1004128.s011.pdf]

**Table S2. Empirical probabilities under neutrality and different selection scenarios**

| | Selection simulation | | | | | | Neutral simulation | |
|---|---|---|---|---|---|---|---|---|
| Recombination Landscape | Constant | | | Hotspot | | | Constant | Hotspot |
| Selection Coefficient | s=0.005 | s=0.01 | s=0.03 | s=0.005 | s=0.01 | s=0.03 | s=0 | s=0 |
| $F_{ST}$ | 0.5400 | 0.5160 | 0.5120 | 0.4800 | 0.5760 | 0.6453 | 0.0020[a] | 0.0020[a] |
| XPEHH | 0.0200 | 0.0036 | 0.0004 | 0.1222 | 0.0276 | 0.0036 | 0.6893 | 0.6964 |
| Fay-WuH | 0.3835 | 0.2059 | 0.0801 | 0.8286 | 0.3878 | 0.0904 | 0.0489 | 0.1071 |
| Fu Li's D | 0.0218 | 0.0120 | 0.0018 | 0.0703 | 0.0218 | 0.0076 | 0.4561 | 0.5006 |
| Tajima's D | 0.0138 | 0.0333 | 0.0240 | 0.1001 | 0.0663 | 0.0374 | 0.2254 | 0.2281 |
| dDAF | 0.5400 | 0.5160 | 0.5120 | 0.4800 | 0.5760 | 0.6440 | 0.0020[a] | 0.0020[a] |
| All statistics combined[b] | 1.3E-06 | 1.5E-07 | 7.8E-10 | 0.0003 | 8.9E-06 | 5. 9E-08 | 6.9E-06 | 1.7E-05 |
| $F_{ST}$-XPEHH-Fay Wu's H | 0.0041 | 0.0004 | 1.8E-05 | 0.0486 | 0.0062 | 0.0002 | 6.7E-05 | 0.0001 |
| $F_{ST}$ -XPEHH-Fu Li's D | 0.0002 | 2.2E-05 | 4.1E-07 | 0.0041 | 0.0003 | 1.7E-05 | 0.0006 | 0.0007 |
| $F_{ST}$ -XPEHH-Tajima's D | 0.0001 | 6.1E-05 | 5.5E-06 | 0.0059 | 0.0011 | 8.6E-05 | 0.0003 | 0.0003 |
| dDAF-XPEHH-Fay Wu's H | 0.0041 | 0.0004 | 1.8E-05 | 0.0486 | 0.0062 | 0.0002 | 6.7E-05 | 0.0001 |
| dDAF-XPEHH-Fu Li's D | 0.0002 | 2.2E-05 | 4.1E-07 | 0.0041 | 0.0003 | 1.7E-05 | 0.0006 | 0.0007 |
| dDAF-XPEHH-Tajima's D | 0.0001 | 6.1E-05 | 5.5E-06 | 0.0059 | 0.0011 | 8.6E-05 | 0.0003 | 0.0003 |

[a] As the simulated distribution did not contain the observed neutrality value, the probability was computed as 1/( number of simulations + 1)

[b] Neutrality statistics include $F_{ST}$ for population differentiation; XPEHH for extended linkage disequilibrium decay; and Tajima's D, Fu Li´s D and Fay Wu's H for site frequency spectrum
