# Supplementary material for: Extreme Population Differences in the Human Zinc Transporter ZIP4 (SLC39A4) Are Explained by Positive Selection in Sub-Saharan Africa
Source: PLoS Genet. 2014 Feb 20;10(2):e1004128. doi: 10.1371/journal.pgen.1004128 (PMC3930504; doi:10.1371/journal.pgen.1004128)
Supplement: Table S3 — Simulation parameters similar to best-fit model Schaffner et al. [31]. (PDF) [file pgen.1004128.s012.pdf]

Table S3. Simulation parameters similar to best-fit model Schaffner et al. [31]

# cosi params file based on bestfit Schaffner et al (2005)	
# in bp.	
length	500000
# per bp per generation	
mutation_rate	1.5e-8
recomb_file	model_Zip4_YRI_hotspot1000g
# observed recombination landscape in YRI population	
# alternative: constant rate calculated as 8.1702e-09	
gene_conversion_rate	4.5e-9
# population info	
pop_define	1 european
pop_define	3 african
#european	
pop_size	1 100000
sample_size	1 170
#african	
pop_size	3 100000
sample_size	3 176
# population events	
pop_event change_size "agriculture - african"	3 200 24000
pop_event change_size "agriculture - european"	1 350 7700
pop_event bottleneck "african bottleneck"	3 3300 .008
pop_event bottleneck "european bottleneck"	1 3301 .02
pop_event bottleneck "OoA bottleneck"	1 3499 .085
pop_event split "out of Africa"	3 1 3500
pop_event change_size "african pop size"	3 17000 12500
#Simulate different selection coefficients in African population	
pop_event sweep "selective sweep"	3 351 .005 .5 .98
#	
