# Supplementary material for: Extreme Population Differences in the Human Zinc Transporter ZIP4 (SLC39A4) Are Explained by Positive Selection in Sub-Saharan Africa
Source: PLoS Genet. 2014 Feb 20;10(2):e1004128. doi: 10.1371/journal.pgen.1004128 (PMC3930504; doi:10.1371/journal.pgen.1004128)
Supplement: Table S4 — Description of primers and hcDNA used in mutagenesis. (PDF) [file pgen.1004128.s013.pdf]

Table S4. Description of primers and hcDNA used in mutagenesis.

Primer A<br>(Val372)	ggcagtgggtgca**gtc**actggggacgctgtcctg
Primer B<br>(Ala357)	ctggctgcaggggggtc**acc**cactacatcctgcagac
Primer C<br>(Pro372)	gcctggcagtgggtgca**ccc**actggggacgctgtc
Primer D<br>(Arg372)	cctggcagtgggtgca**cgc**actggggacgctgtc
hcDNA_cloned<br><br>(Thr357-Leu372)	atggcgtccctggtctcgctggagctggggctgcttctggctgtgctggtggtgacggcgacggcgtccccgcctgctggtctg<br>ctgagcctgctcacctctggccagggcgctctggatcaagaggctctgggcggcctgttaaatacgctggcggaccgtgtgc<br>actgcaccaacgggccgtgtggaaagtgcctgtctgtggaggacgccctgggcctgggcgagcctgaggggtcagggctgc<br>ccccgggcccggtcctggaggccaggtacgtcgcccgcctcagtgccgccgccgtcctgtacctcagcaaccccgagggcac<br>ctgtgaggacactcgggctggcctctgggcctctcatgcagaccacctcctggccctgctcgagagccccaaggccctgacc<br>ccgggcctgagctggctgctgcagaggatgcaggcccgggctgccggccagacccccaagacggcctgcgtagatatccct<br>cagctgctggaggaggcggtgggggcgggggctccgggcagtgctggcggcgtcctggctgccctgctggaccatgtcagg<br>agcgggtcttgcttccacgccttgccgagccctcagtacttcgtggactttgtgttccagcagcacagcagcgaggtccctatg<br>acgctggccgagctgtcagccttgatgcagcgcctgggggtgggcagggaggcccacagtgaccacagtcatcggcacagg<br>ggagccagcagccgggaccctgtgcccctcatcagctccagcaacagctccagtgtgtgggacacggtatgcctgagtgcc<br>agggacgtgatggctgcatatggactgtcggaacaggctggggtgaccccggaggcctgggcccaactgagccctgccctg<br>ctccaacagcagctgagtggagcctgcacctcccagtccaggcccccgtccaggaccagctcagccagtcagagaggtat<br>ctgtacggctccctggccacgctgctcatctgcctctgcgcggtctttggcctcctgctgctgacctgcactggctgcaggggg<br>gtc**gcc**cactacatcctgcagaccttcctgagcctggcagtgggtgca**ctc**actggggacgctgtcctgcatctgacgcccaa<br>ggtgctggggctgcatacacacagcgaagagggcctcagcccacagcccacctggcgcctcctggctatgctggccgggct<br>ctacgccttcttcctgtttgagaacctcttcaatctcctgctgcccagggacccggaggacctggaggacgggccctgcggcc<br>acagcagccatagccacgggggccacagccacggtgtgtccctgcagctggcacccagcgagctccggcagcccaagccc<br>ccccacgagggctcccgcgcagacctggtggcggaggagagcccggagctgctgaaccctgagcccaggagactgagccc<br>agagttgaggctactgccctatatgatcactctgggcgacgccgtgcacaacttcgccgacgggctggccgtgggcgccgcc<br>ttcgcgtcctcctggaagaccgggctggccacctcgctggccgtgttctgccacgagttgccacacgagctgggggacttcgc<br>cgccttgctgcacgcggggctgtccgtgcgccaagcactgctgctgaacctggcctccgcgctcacggccttcgctggtctcta<br>cgtggcactcgcggttggagtcagcgaggagagcgaggcctggatcctggcagtggccaccggcctgttcctctacgtagca<br>ctctgcgacatgctcccggcgatgttgaaagtacgggacccgcggccctggctcctcttcctgctgcacaacgtgggcctgct<br>gggcggctggaccgtcctgctgctgctgtccctgtacgaggatgacatcaccttc
